# Receptor tyrosine kinase EphA7 is required for interneuron connectivity at specific subcellular compartments of granule cells

**DOI:** 10.1038/srep29710

**Published:** 2016-07-13

**Authors:** Simone Beuter, Ziv Ardi, Omer Horovitz, Jennifer Wuchter, Stefanie Keller, Rinki Saha, Kuldeep Tripathi, Rachel Anunu, Orli Kehat, Martin Kriebel, Gal Richter-Levin, Hansjürgen Volkmer

**Affiliations:** 1 Dept. Molecular Biology, Natural and Medical Sciences Institute at the University of Tübingen, Markwiesenstr. 55, 72770 Reutlingen, Germany; 2 Sagol Department of Neurobiology, University of Haifa, Mount Carmel, 31095, Israel; 3 The Institute for the Study of Affective Neuroscience (ISAN), University of Haifa, Mount Carmel, 31095, Israel; 4 Department of Psychology, University of Haifa, Mount Carmel, 31095, Israel

## Abstract

Neuronal transmission is regulated by the local circuitry which is composed of principal neurons targeted at different subcellular compartments by a variety of interneurons. However, mechanisms that contribute to the subcellular localisation and maintenance of GABAergic interneuron terminals are poorly understood. Stabilization of GABAergic synapses depends on clustering of the postsynaptic scaffolding protein gephyrin and its interaction with the guanine nucleotide exchange factor collybistin. Lentiviral knockdown experiments in adult rats indicated that the receptor tyrosine kinase EphA7 is required for the stabilisation of basket cell terminals on proximal dendritic and somatic compartments of granular cells of the dentate gyrus. EphA7 deficiency and concomitant destabilisation of GABAergic synapses correlated with impaired long-term potentiation and reduced hippocampal learning. Reduced GABAergic innervation may be explained by an impact of EphA7 on gephyrin clustering. Overexpression or ephrin stimulation of EphA7 induced gephyrin clustering dependent on the mechanistic target of rapamycin (mTOR) which is an interaction partner of gephyrin. Gephyrin interactions with mTOR become released after mTOR activation while enhanced interaction with the guanine nucleotide exchange factor collybistin was observed in parallel. In conclusion, EphA7 regulates gephyrin clustering and the maintenance of inhibitory synaptic connectivity via mTOR signalling.

The local circuitry plays an important role in the control of excitatory principal neurons in the brain. In this context, different classes of GABAergic interneurons target axonal, somatic or dendritic compartments of principal neurons and thereby might differentially contribute to the control of neuronal transmission^1^,^2^. Presently, only little information is available on how terminals of different types of interneurons are stabilised at different neuronal compartments of postsynaptic cells. In general, the stabilisation of GABAergic synapses relies on the postsynaptic scaffolding protein gephyrin, which needs to be clustered at the postsynaptic membrane for proper alignment of GABA_A_ receptors^3^,^4^. Gephyrin translocation to the membrane and its clustering depend on the nucleotide exchange factor collybistin^5^,^6^,^7^,^8^. Upstream, gephyrin clustering is further controlled by growth factors activating mitogen-activated protein kinase (MAPK) and phosphatidylinositol-4,5-bisphosphate 3-kinase (PI3K)-Akt pathways^9^. In particular, the Akt target glycogen synthase kinase 3β (GSK3β) as well as extracellular-signal regulated kinase (Erk) phosphorylate gephyrin at serine-270 and serine-268, respectively, and thereby regulate gephyrin clustering^10^,^11^. Control of gephyrin clustering is additionally achieved via interactions with mechanistic target of rapamycin (mTOR)^9^. Altogether, these findings suggest common mechanisms for gephyrin clustering, which raises the question how gephyrin and GABA_A_ receptor clustering as well as concomitant stabilisation of inhibitory synapses are ensured at specific subcellular compartments. Possibly, expression of upstream cell surface receptors governs the selective activation of gephyrin clustering at specific subcellular sites.

Considering candidate receptors involved in the subcellular control of gephyrin clustering, we regarded EphA7 as an interesting candidate expressed on dendritic and somatic compartments of granule cells in the hippocampus^12^. EphA7 is a member of a subclass of eight receptor tyrosine kinases interacting with five different ephrin A ligands in a highly promiscuous way^13^. Ephrin ligands are membrane-bound, suggesting a role in cell-cell interaction and trans-synaptic signalling. Accordingly, ephrin-Eph interactions were proposed to regulate synaptic plasticity although mainly functions for topographic projection in neurodevelopment have been assigned (for review see ref. 14). Here, we identify EphA7 as a cell surface receptor specifically required for the stabilisation of synaptic terminals of parvalbumin-positive basket cells targeting somata and proximal dendrites of granule cells, the principal neurons of the rat dentate gyrus. Specifically, knockdown of EphA7 in adult animals provoked a specific loss of basket cell innervation and impaired long-term potentiation (LTP) and hippocampal learning. Finally, we provide evidence that EphA7 induces gephyrin clustering via PI3K-Akt-mTOR signalling.

## Results

### EphA7 expression is required for the stabilisation of basket cell terminals *in vivo*

**Figure 1:**
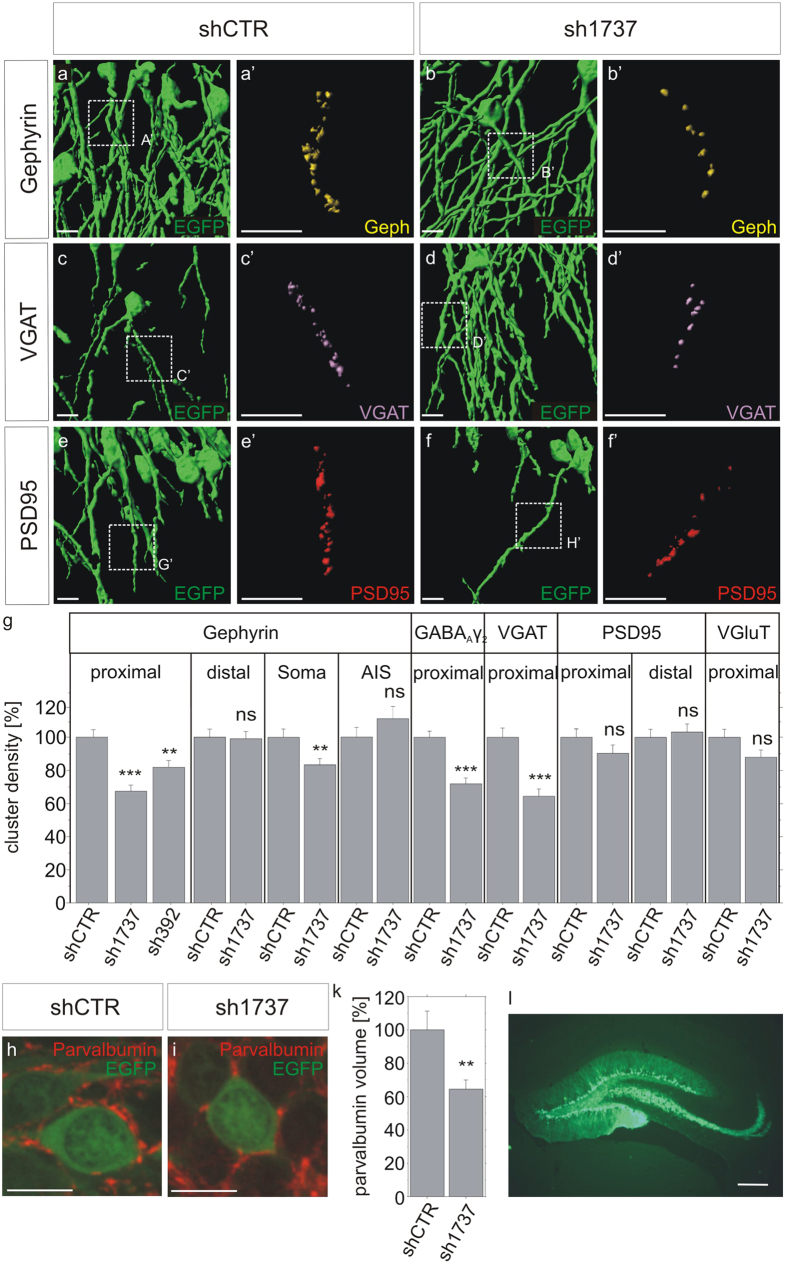
EphA7 is required for the stabilisation of GABAergic synaptic markers at proximal dendrites and somata of granule cells *in vivo*. (**a–g**) Lentiviral vectors were stereotaxically injected into the dentate gyrus of adult female rats. For the identification of transduced principal neurons, lentiviral constructs expressed EGFP under the control of the CamKII promoter. Likewise, EphA7-specific shRNA 1737 or sh392 were coexpressed from the same vector as indicated. Brain slices were stained for pre- and postsynaptic, inhibitory and excitatory synapse markers VGAT, gephyrin, VGluT, PSD95, and the γ2 subunit of GABA_A_ receptors, respectively. Example micrographs are shown in a-f. Insets in (**a–f**) are enlarged in (a’–f’). All images were rendered using IMARIS software. Scale bars, 10 μm. (**g**) Quantification of synaptic marker densities on different compartments of granule cells as indicated. Proximal dendritic segments: gephyrin, n = 140, ***p < 0.0001; GABA_A_ γ2, n = 40, ***p < 0.0001; VGAT, PSD95, VGluT, n = 90, ***p < 0.0001; ns, not significant. Distal dendritic segments: gephyrin, PSD95, n = 70; ns, not significant. Soma: gephyrin, n = 60, **p < 0.01. Axon initial segment (AIS): gephyrin, n = 60; ns, not significant. ANOVA, Fisher-PLSD; error bars: S.E.M. (**h,i**) Representative images of granule cells transduced with control or EphA7 knockdown lentivirus coexpressing EGFP and stained for parvalbumin. (**k**) Parvalbumin-stained voxels representative for basket cell terminals were identified and quantified as objects in confocal z-stacks. The volume of parvalbumin-positive structures per contacted soma were determined as a measure for basket cell innervation after EphA7 knockdown (65%) compared to control (100%). n = 26, **p < 0.01; ANOVA, Fisher-PLSD; error bars: S.E.M. scale bars: 10 μm. (**l**) Representative image of EGFP fluorescence indicating lentiviral infection of the dentate gyrus. Scale bar 200 μm.

EphA7 was previously shown to be expressed in dendritic fields of granule cells of the dentate gyrus^12^. In a more precise analysis, EphA7 staining was identified on proximal dendritic segments and on somata of granular cells, a subcellular compartment targeted by inhibitory basket cells (supplementary Figure S1). At higher magnifications, clusters of gephyrin, a marker for inhibitory postsynapses, also colocalized with EphA7 clusters on somata and dendrites of cultured neurons (supplementary Figure S1c). Therefore, lentiviral shRNA vectors were constructed and injected into the rat dentate gyrus in order to study the impact of reduced EphA7 expression on synaptic input onto granule cells *in vivo*. For the exclusion of off-target effects, two independent lentiviral knockdown vectors were applied expressing two shRNAs specific for EphA7 mRNA (sh392 and sh1737). As a control, an ineffective shRNA vector (shCTR) was included. Lentiviral vectors coexpressed EGFP under the control of the CamKII-promoter for the identification of single infected neurons. Partially reduced EphA7 mRNA expression was confirmed by quantitative RT-PCR in hippocampal neurons after infection with lentiviral vectors sh392 and sh1737 *in vitro* while decreased EphA7 protein levels were assessed in single granule cells after stereotaxic injection of lentiviral shRNA vectors *in vivo* (supplementary Figure S2). Two weeks after injection, densities of synaptic markers at different subcellular compartments of EGFP-positive infected granule cells were determined (Fig. 1a–g,l). Gephyrin and VGAT were chosen as post- or presynaptic markers for GABAergic synapses while PSD95 and VGlut were used for glutamatergic synapses, respectively. Injection of lentiviral vectors sh392 and sh1737 both significantly reduced gephyrin cluster densities in proximal dendritic segments granule cells (sh1737- 68% and sh392 - 83% of the control, Fig. 1g). Since reduced cluster densities of γ2 subunits of GABA_A_ receptors (70% of control) and of presynaptic VGAT (64% of control) were observed in parallel, these results also suggest a loss of inhibitory terminals on proximal dendritic segments. Lentiviral vector sh1737 also decreased gephyrin cluster densities on somata (84% of control) while no significant changes were observed in axon initial and distal dendritic segments. Cluster densities of PSD95 and VGluT remained unaffected, suggesting that EphA7 knockdown does not affect the maintenance of excitatory synapses. Application of both vectors sh392 and sh1737 also decreased gephyrin cluster densities in cultured neurons (supplementary Figure S2).

Parvalbumin-positive basket cells were previously shown to target proximal dendrites and somata of granule cells in the dentate gyrus^15^. The locally restricted loss of GABAergic terminals after EphA7 knockdown suggested a requirement of EphA7 expression for the stabilization of basket cell terminals. Therefore, dentate gyrus slice preparations were further stained with an antibody specific for the basket cell marker parvalbumin in order to confirm an impact of EphA7 on the stabilisation of basket cell terminals on granule cells (Fig. 1h,i). The volume of parvalbumin-positive structures attributed to basket cell terminals was localised on EGFP-positive granule cell somata and taken as a measure for basket cell innervation after infection with an EphA7 knockdown vector. Quantitative analysis showed that basket cell innervation was significantly decreased after EphA7 knockdown in comparison to the control situation (65% of the control, Fig. 1k, p < 0.01). These results suggest that local expression of EphA7 on granule cells in the differentiated dentate gyrus is required for the stabilisation of basket cell terminals.

In conclusion, the observed decrease in inhibitory pre- and postsynaptic markers implies that EphA7 expression is specifically required for the stabilisation of inhibitory parvalbumin-positive basket cell terminals on proximal dendritic segments and on somata of granule cells. It is of note that EphA7 now appears as a spatial regulator in sub-compartments within a single cell in our approach while previous findings understood EphA7 as a compartmentalizing factor at the tissue level^16^,^17^.

### LTP induction and hippocampus-dependent learning are impaired by EphA7 knockdown

**Figure 2:**
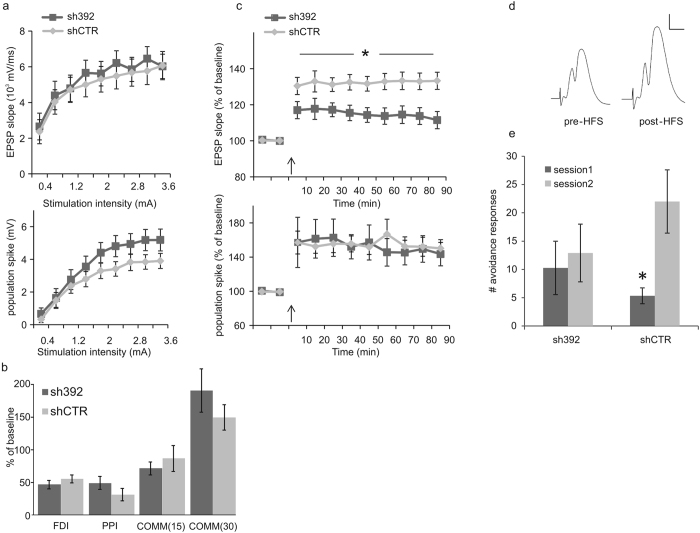
EphA7 knockdown rats impairs long-term potentiation and learning. (**a**) Basic excitability parameters of the dentate gyrus neuronal population (field recordings). Intrinsic excitability of the neurons (i.e. population spike; top) and synaptic excitability (i.e. EPSP slope; bottom) are enhanced with increasing stimulation amplitude applied from the perforant path. EphA7 knockdown group: n = 12; control group: n = 11. No significant differences were found between groups (p > 0.05). Error bars, S.E.M. (**b**) Alterations in population spikes following frequency-dependent inhibition (FDI; stimulation frequency was increased from 0.1 to 1 Hz), paired-pulse inhibition (PPI; interval between the two pulses was 15 ms), and commissural inhibition (COMM; commissural pulse preceded perforant path pulse with 15/30 ms). Data are expressed as percentage of baseline measurements, averaged for 5 (PPI and COMM) or 10 (FDI) pulses. Knockdown group: n = 12; control group: n = 11. No significant differences were found between groups (p > 0.05). Error bars: S.E.M. (**c**) LTP following high frequency stimulation. Potentiation of the EPSP slope (top) and population spike (bottom) following theta burst stimulation, expressed as percentage of baseline measurements. The knockdown group (n = 9) exhibits reduced slope of the EPSP in comparison to the control group (n = 8). Arrows indicate time of high frequency stimulation. *p < 0.05; error bars, S.E.M. (**d**) Example traces of field recordings from a control rat before (left) and after (right) theta burst stimulation. Calibration: 5 ms/2 mV. (**e**) Avoidance responses per session in the two way shuttle avoidance task. Control group (n = 11) improves performance in the second session, while knockdown group (n = 12) shows no such improvement. *p < 0.01; error bars: S.E.M. ANOVA.

From the experiments presented above, knockdown of EphA7 evolved as a suitable tool to specifically remove basket cell terminals from granule cells of the dentate gyrus. We therefore conducted a further set of experiments to analyze the physiological impact of EphA7 knockdown. To this end, lentiviral EphA7 knockdown vectors were injected into the dentate gyrus of adult rats. Subsequently, animals were examined using field potential recordings in the dentate gyrus under anesthesia *in vivo*. Finally, the site of injection in the dentate gyrus was verified by microscopic analysis of EGFP fluorescence in brain. First, intrinsic excitability of the neurons (i.e. the population spike) and synaptic excitability (i.e. EPSP-slope) were measured at increasing stimulation amplitudes applied to the perforant path. No significant difference between EphA7 knockdown and control rats was observed in these measurements (Fig. 2a).

Inhibitory synapses formed between local interneurons and principal neurons represent basic elements of local circuitry. Since EphA7 is specifically involved in basket cell innervation, which is part of the local circuitry, we next analyzed the impact of EphA7 knockdown on local circuit activity in the dentate gyrus using three different stimulation protocols including frequency-dependent inhibition (FDI), paired pulse inhibition (PPI), and commissural modulation inhibition (COMM, 15 or 30 ms, respectively). Both sh392 and shCTR-treated groups showed alterations in population spikes in comparison to baseline recordings, but no significant differences were found in the extent of inhibition between both groups (Fig. 2b). Therefore, EphA7 knockdown in the dentate gyrus did not appear to be involved in these particular types of feed-back and feed-forward inhibition, which discriminates basket cell functions from chandelier cell activities required for feed-back inhibition via axo-axonic synapses^18^.

In a further set of experiments, we analyze LTP in granule cells of the dentate gyrus after perforant path stimulation. Potentiation of the population spike and the EPSP-slope following theta-burst stimulation was recorded for 90 minutes and expressed as percentage of baseline measurements. Knockdown of EphA7 significantly reduced the level of potentiation of the EPSP slope (p < 0.05, repeated measures ANOVA) in comparison to the control group, but did not influence the level of potentiation in the population spike (Fig. 2c,d). Therefore, EphA7 knockdown and concomitant reduction in basket cell innervation account for a significant reduction in LTP.

Considering the role of the hippocampus both in memory formation and stress response regulation, we sought out to assess EphA7 involvement in these processes. We therefore examined whether EphA7 knockdown within the dentate gyrus is sufficient to alter anxiety-related behaviors in the open field and elevated plus maze and in learning under stressful conditions in the two-way shuttle avoidance task. Results showed no significant difference between control and knockdown rats in time spent in the periphery and in the center of the open field, nor in the locomotor activity in both areas of the open field. In addition, in the elevated plus maze test, no significant differences were found between the two groups in the time spent in the open arms in comparison to the closed arms or the locomotor activity in either arm (data not shown). Thus, no differences in anxiety levels were present when comparing the two groups. Learning under stressful conditions was examined in the two way shuttle avoidance task. Rats underwent two training sessions on two consecutive days and performance on the task was measured by the number of avoidance responses. Results revealed a significant main effect for session (p < 0.01) and a significant group/session interaction (p < 0.05, factorial repeated measures ANOVA). Further testing showed that control rats improved their performance in the second training session in comparison to the first (p < 0.01, paired test), while knockdown rats showed no such improvement (Fig. 2e). In summary, our data indicate that EphA7 knockdown and concomitant loss of basket cell innervation correlates with reduced LTP and hippocampal learning.

### EphA7 induces gephyrin clustering depending on its protein kinase activity

**Figure 3:**
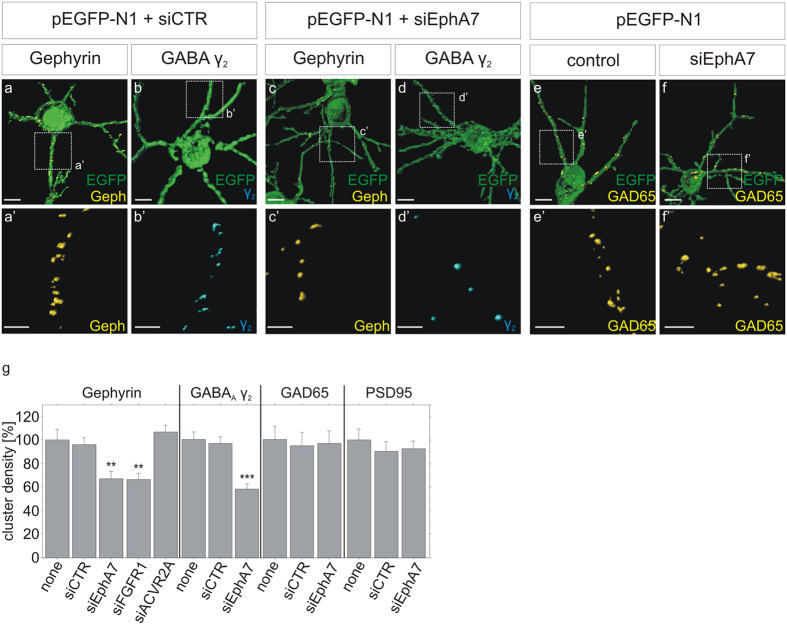
siRNA-mediated knockdown of EphA7 reduces gephyrin cluster density in hippocampal neurons. (**a–g**) Hippocampal neurons were cotransfected with pEGFP-N1, a scrambled control siRNA (siCTR) or siRNA specific for EphA7 (siEphA7), for FGFR1 (siFGFR1), or for ACVR2A (siACVR2). Cultures were fixed and stained for expression of gephyrin (**a,c**), the γ2 sub-unit of GABA_A_ receptors (**b,d**) or GAD65 (**e,f**). Transfected cells were identified by green EGFP fluorescence. Magnifications of insets in (**a–f**) are shown in (a’–f’). All images were rendered using Imaris software. Scale bars, 10 μm. (**g**) Densities of gephyrin, GAD65, PSD95 and γ2 subunit punctae were quantified per 20 μm dendritic segment, n = 30; **p < 0.01; ***p < 0.0001; ANOVA, Fisher-PLSD. For knockdown efficiency of EphA7 siRNA see supplementary Figure S2.

**Figure 4:**
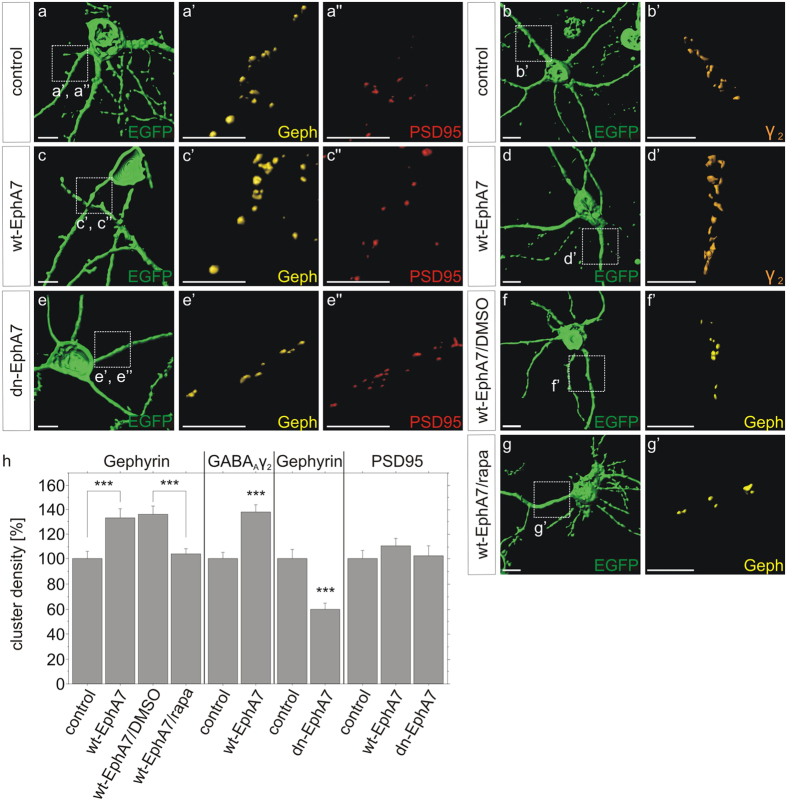
Overexpression of wild type EphA7 or stimulation with ephrin A5 increases while protein kinase-deficient EphA7 reduces gephyrin clustering. (**a,c,e**) After cotransfection of pEGFP-N1 and wildtype EphA7 (wt-EphA7) or dominant-negativ EphA7 (dn-EphA7), cells were stained for gephyrin (yellow, a’,c’, e’) or PSD95 (red, a”,c”, e”). Magnifications of insets in (a-g) are shown in (a’–g’), and in (a”–e”), respectively. All images were rendered using Imaris software. Scale bars, 10 μm. (**b,d**) control and wt-EphA7 transfected cells were stained for the γ2 subunit of GABA_A_ receptors. (**f,g**) Neurons transfected with wt-EphA7 were treated with DMSO or rapamycin/DMSO (rapa) and subsequently stained for gephyrin. (**h**) The densities of gephyrin, γ2 subunit of GABA_A_ receptors and PSD95 punctae were quantified in transfected neurons as identified by EGFP fluorescence. wt-EphA7: gephyrin, n = 50; GABA_A_ γ2, n = 45; PSD95, n = 30. dn-EphA7: gephyrin, n = 30, ***p < 0.0001, ANOVA, Fisher-PLSD. Error bars, S.E.M.

Postsynaptic scaffolding molecule gephyrin was shown to interact with and assemble GABA_A_ receptors in the postsynaptic membrane and thereby contribute to the dynamics or stabilisation of inhibitory synapses^19^. We hypothesized EphA7 signalling as an underlying mechanism involved in gephyrin clustering and concomitant stabilisation of GABAergic synapses. We tested this assumption in cultured primary neurons which provide a valid model to study cell-autonomous mechanisms for postsynaptic organisation^11^,^20^,^21^. First of all, siRNA transfection was applied to confirm a specific link between EphA7 expression and gephyrin clustering in cultured neurons. Similar to shRNA knockdown, alternative transfection of EphA7-specific siRNA reduced gephyrin and GABA_A_ receptor clustering in hippocampal neurons (Fig. 3). Knockdown of FGFR1 (fibroblast growth factor signalling) or ACVR2A (TGFβ signalling) served as a positive or negative control, respectively. No impact was observed on excitatory synapse marker PSD95 or the presynaptic GAD65 marker. Efficient siRNA-mediated knockdown of EphA7 at the mRNA and the protein level is shown in supplemental Figure S2. Supplementing the loss-of-function experiments, we analyzed the impact of EphA7 overexpression on gephyrin clustering in a gain-of-function experiment. After transfection of a wild type EphA7 expression vector into hippocampal neurons (Fig. 4a–g), gephyrin cluster densities at dendritic segments were significantly increased (133%) as compared to control transfected neurons indicating that EphA7 expression was sufficient to induce gephyrin clustering (Fig. 4h; p < 0.0001). Increased EphA7 expression after transfection is shown in supplemental Figure S3. The increase in gephyrin cluster density was reduced to basal levels by application of the mTOR inhibitor rapamycin, suggesting a possible link between mTOR and EphA7 in accordance with a recent report^22^. Parallel analysis of γ2 subunits of GABA_A_ receptor clusters showed enhanced cluster density (139% of the control) supporting the view that gephyrin clustering is intimately linked to the distribution of GABA_A_ receptors. In contrast, overexpression of wild type EphA7 did not increase gephyrin cluster size, significantly (supplemental Figure S3). We further asked whether EphA7 kinase activity is required for the stabilisation of gephyrin clusters. A point mutation was introduced into the EphA7 cDNA sequence to provide the dominant-negative K665M mutation (dn-EphA7) in the protein kinase sequence of human EphA7^23^. Transfection and overexpression of dn-EphA7 significantly reduced gephyrin cluster density (Fig. 4h; 60% of the control, p < 0.0001), implying that the protein kinase activity of EphA7 is required for gephyrin clustering. No effects on PSD95 clustering were observed after expression of both constructs, confirming a selective impact on postsynaptic scaffold proteins of inhibitory synapses in accordance with the *in vivo* experiments. Hence, EphA7 expression appeared to be necessary and sufficient for gephyrin clustering.

### Ephrin A5 stimulation increases gephyrin clustering

**Figure 5:**
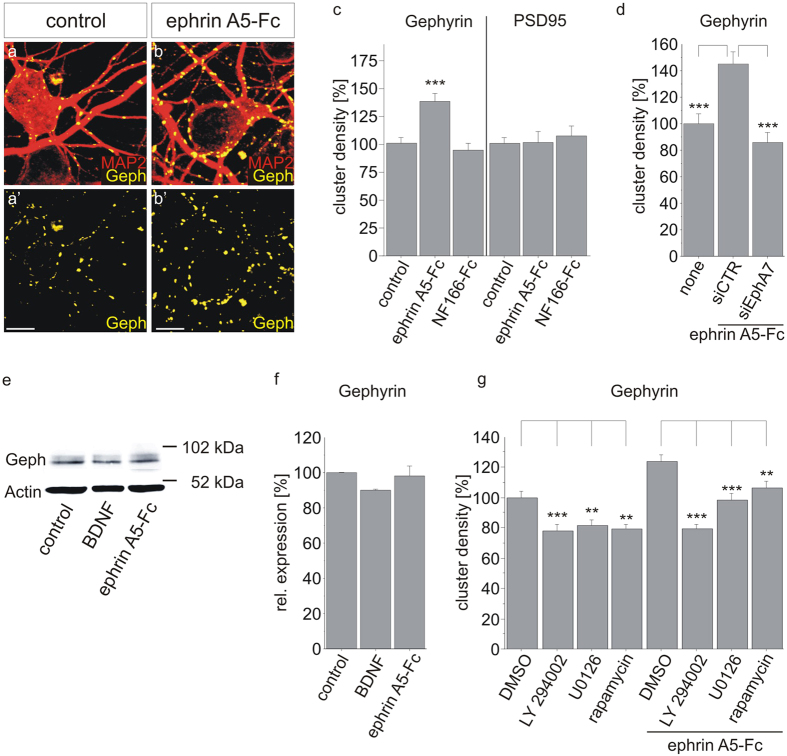
Ephrin A5 stimulation increases gephyrin cluster density via EphA7. (**a,b**) Hippocampal cultures were treated with preclustered ephrin A5-Fc for 24 hrs. Cells were co-stained for MAP2 (red) and gephyrin (yellow). (a’, b’) The same micrographs as in (**a,b**), showing gephyrin signals only. All images were rendered using Imaris software. Scale bars, 10 μm. (**c**) After ephrin A5-Fc treatment, gephyrin and PSD95 cluster densities were quantified on MAP2-positive dendrites. n = 30; ***p < 0.001. (**d**) Prior to ephrin A5-Fc stimulation, hippocampal neurons were transfected with control siRNA or siRNA specific for EphA7 as well as pEGFP-N1 for the identification of transfected cells. n = 30; ***p < 0.0001. (**e,f**) Determination of relative gephyrin expression levels after ephrin A5-Fc treatment. Protein lysates of cortical neurons were treated for 30 min with BDNF or preclustered ephrin A5-Fc, submitted to SDS-PAGE and subsequent Western Blotting. Gephyrin expression was quantified in comparison to the actin signal as a loading control. n = 3. (**g**) Untreated or ephrin A5-Fc-stimulated hippocampal neurons were further treated with inhibitors for PI3K (Ly294002), MEK (U0126), or mTOR (rapamycin) for 24 hrs. n = 135; **p < 0.01; ***p < 0.0001; ANOVA, Fisher-PLSD; error bars, S.E.M.

Since EphA7 is a target for ephrin A ligands, ephrin A stimulation is expected to induce gephyrin clustering^13^,^24^. We chose ephrin A5 stimulation since it was shown to be expressed in the postnatal hippocampus^25^. Furthermore, ephrin A5 has been shown to interact with EphA7 while ephrin A5-EphA7 interactions contribute to dendritic maturation^16^,^22^. Recombinant ephrin A5 fused to the Fc-portion of IgG (ephrin A5-Fc) was pre-clustered with an anti-IgG antibody and applied to hippocampal neurons at a concentration of 100 ng/ml (Fig. 5a,b). Ephrin A5-Fc significantly increased gephyrin cluster density (141% of the control, p < 0.0001) after 24 hrs of incubation while application of an irrelevant control fusion protein (neurofascin-Fc) remained ineffective (Fig. 5c). Ephrin A5-Fc did not alter PSD95 clustering, further confirming specificity for inhibitory postsynapses. Since ephrin A5 may interact with different EphA receptors^26^, siRNA specific for EphA7 as well as a control siRNA were transfected into cultured hippocampal neurons before ephrin A5-Fc stimulation in order to provide evidence for a contribution of EphA7 (Fig. 5d). Ephrin A5-induced gephyrin clustering was reduced to basal levels by EphA7-specific siRNA, indicating that EphA7 represents a target of ephrin A5. Increased mTOR activity may contribute to enhanced translation of gephyrin^27^. However, ephrin A5-Fc application did not induce gephyrin expression at the protein level (Fig. 5e,f). Therefore, increased gephyrin clustering most probably relies on increased clustering of already existing gephyrin molecules and not on increased expression.

Gephyrin clustering was shown to be regulated by BDNF-trkB and downstream signalling components including the MAPK pathway and the PI3K-Akt-mTOR pathway^9^. We therefore investigated a possible contribution of MAPK and PI3K-Akt-mTOR pathways to EphA signalling and its impact on gephyrin clustering. Hippocampal neurons were stimulated with ephrin A5-Fc in the absence or presence of inhibitors for MEK (U0126), PI3K (Ly294002) as well as for mTOR (rapamycin, Fig. 5g). All three inhibitors decreased baseline and ephrin A5-induced gephyrin cluster density. In conclusion, ephrin A5 stimulation increased gephyrin clustering in accordance with an involvement of EphA signalling. Likewise, application of inhibitors point at a possible contribution of the PI3K-mTOR pathway in EphA-induced gephyrin clustering.

### Akt and mTOR become phosphorylated upon ephrin A5 stimulation

**Figure 6:**
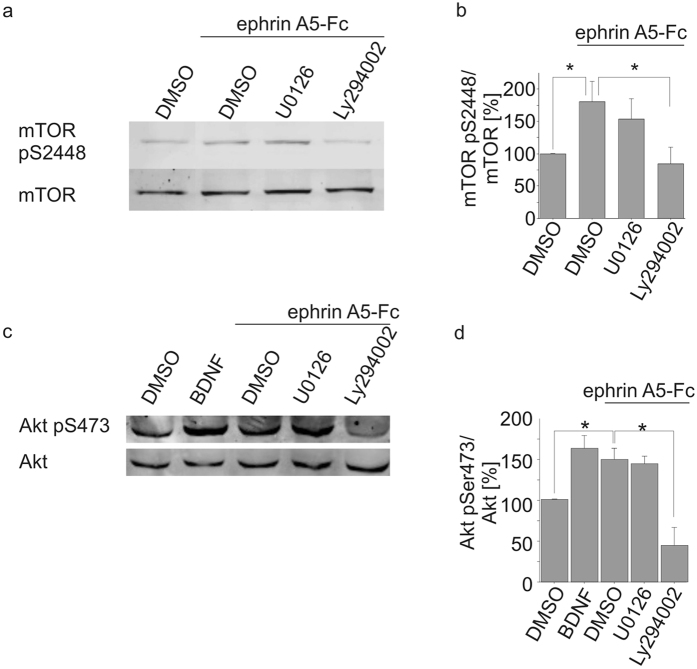
Ephrin A5-Fc stimulation induces mTOR phosphorylation. Protein lysates of cortical neurons treated with BDNF or ephrin A5-Fc as well as inhibitors for PI3K (Ly294002) or MEK (U0126) were submitted to SDS-PAGE and Western blotting. mTOR (**a,b**), ERK1/2 and Akt1 (**c,d,e**). Protein expression was determined by densitometric analysis of the corresponding protein bands. Phosphorylation was quantified and calculated as ratios of signals obtained with antibodies specific for pan-mTOR and phospho-mTOR (mTOR pS2448) or pan-Akt1 and phosphor-Akt1 (pS473). For mTOR: n = 5; for Akt1 n = 3. ANOVA, Fisher-PLSD; *p < 0.05 **p < 0.01; error bars: S.E.M.

For further confirmation, the phosphorylation and activation of mTOR was studied after ephrin A5 stimulation. After 30 min of ephrin A5-Fc treatment of cultured neurons, S6 kinase (S6K), a down-stream target of activated mTOR has become phosphorylated whereas long term stimulation for 24 hrs was ineffective (supplemental Figure S4). Western blot analysis revealed that ephrin A5 stimulated phosphorylation of serine-2448 of mTOR (180% of the control, p < 0.05; Fig. 6a,b). mTOR phosphorylation was reduced to base line after treatment with the PI3K inhibitor Ly294002 (p < 0.05), further supporting a contribution of PI3K for ephrin A5-induced mTOR phosphorylation, while MEK inhibitor U0126 was ineffective. Ephrin-A5 treatment also stimulated S473 phosphorylation of Akt which is an mTORC2 substrate (Fig. 6c,d). Akt phosphorylation was decreased by PI3K inhibitor Ly294002. In summary, ephrin A5 stimulation *in vitro* induced a significant increase in mTOR phosphorylation. Combined with the data presented in Fig. 4, our results suggest that EphA signalling induced gephyrin clustering through mTOR.

### mTOR activation is sufficient to induce gephyrin clustering

**Figure 7:**
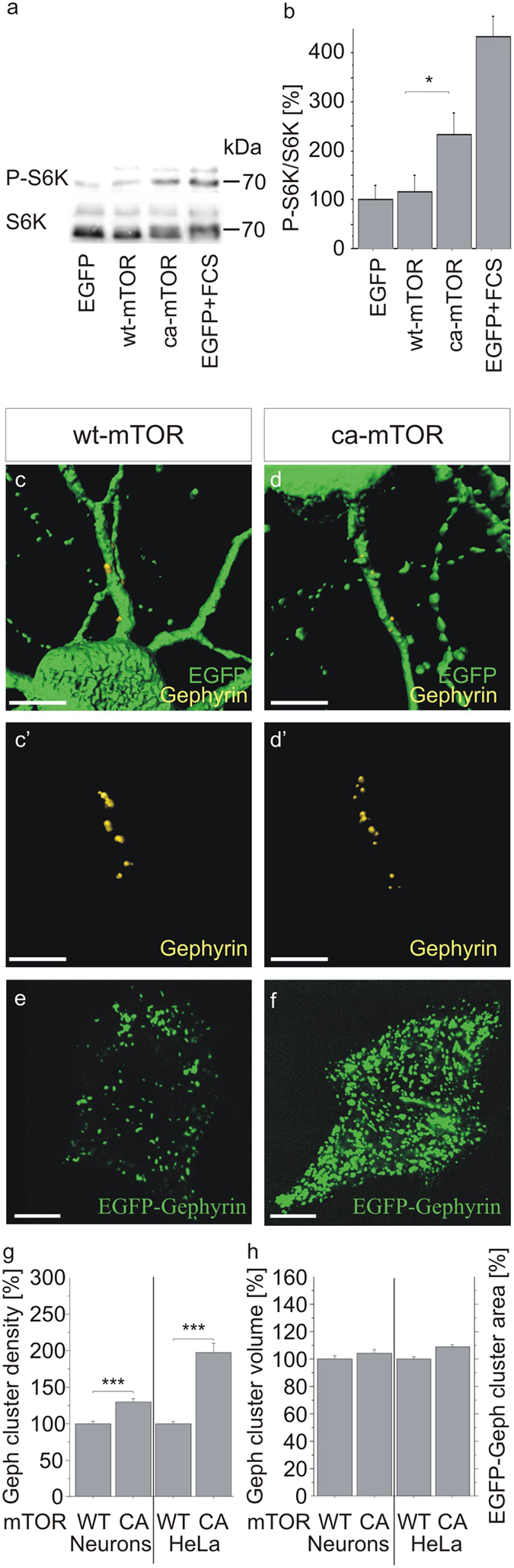
mTOR activation increases gephyrin clustering. (**a,b**) ca-mTOR induces S6K phosphorylation. Lysates of HeLa cells transfected with plasmid vectors expressing EGFP, wt-mTOR, or ca-mTOR were analyzed by Western blotting to quantify total S6K protein (70 kDa isoform) or phosphor-S6K. Stimulation with fetal calf serum (FCS) served as a control. S6K proteins were detected by a pan-S6K antibody (S6K) or a phospho-S6K antibody (pS6K; pThr389), respectively (upper bands correspond to the 85 kDa isoform of S6K). (**b**) Densitometric analysis of signals obtained after western blotting as exemplified in (**a**). Bar charts represent ratios of pS6K to pan-S6K signals as percentage of the EGFP-transfected control. n = 6; *p < 0.05. ANOVA, Fisher-PLSD; error bars, S.E.M. (**c**) ca-mTOR increases gephyrin cluster densities. Either hippocampal neurons (**c,d**); gephyrin staining in c,d is shown in c’,d’) or HeLa cells (**e,f**) were transfected with plasmid vectors for wild type mTOR (WT) or ca-mTOR (CA). Neurons were cotransfected with pEGFP-N1 for the identification of mTOR transfected cells whereas HeLa cells were additionally cotransfected with EGFP-gephyrin and collybistin expression plasmids to allow for surface translocation of expressed EGFP-gephyrin. Cluster densities of immunocytochemically labeled endogenous gephyrin on dendritic segments of EGFP-positive neurons (n = 40) or of EGFP-gephyrin (n = 13) on the surface of HeLa cells were determined. Values are indicated as percentage of wt-mTOR transfected cells. ***p < 0.0001; Kruskal-Wallis-Test, followed by Dwass-Steel-Test; error bars, S.E.M. (**d**) Gephyrin cluster size was determined in primary neurons (cluster volumes) or in HeLa cells (surface cluster areas) after transfection of expression vectors for wt-mTOR or ca-mTOR. n > 13; ***p < 0.0001. ANOVA, Fisher-PLSD; error bars, S.E.M. scale bars 30 μm.

In further experiments, the role of mTOR activation for gephyrin clustering was examined more closely. We constructed an expression plasmid for constitutively active mTOR (ca-mTOR) based on the mutation S2215Y found in a tumour patient^28^. The phenotype of ca-mTOR was verified after transfection into HeLa cells and subsequent analysis of S6 kinase phosphorylation (S6K), a down-stream target of mTOR (Fig. 7a,b). Transfection of an EGFP expressing plasmids or stimulation with serum served as negative or positive controls, respectively. Phosphorylation of S6K was significantly increased in the presence of ca-mTOR (233%) as compared to the negative control (100%) and wildtype mTOR (wt-mTOR; 114%) demonstrating the constitutively active phenotype of ca-mTOR in HeLa cells. Long term expression of ca-mTOR in transfected cultured neurons reduced S6K phosphorylation suggesting negative feedback mechanisms (supplemental Figure S4). Next, wt-mTOR and ca-mTOR were transfected into primary neurons and clustering of endogenous gephyrin was analyzed by quantitative immunocytochemistry (Fig. 7c,d). In comparison to wt-mTOR (100%), expression of ca-mTOR significantly increased gephyrin cluster density (126%) on dendritic segments while cluster volume remained unaffected (p < 0.0001; Fig. 7g,h). The same experiment was conducted in HeLa cells after cotransfection of wt-/ca-mTOR, EGFP-gephyrin and collybistin (Fig. 7e,f). We applied collybistin II SH3- that translocates gephyrin to the membrane in the absence of further components^6^. Accordingly, EGFP-gephyrin cluster density was increased by ca-mTOR (196%) while no effects were observed regarding the size of EGFP-gephyrin cluster areas. Therefore, activation of mTOR is sufficient for the induction of gephyrin clustering.

**Figure 8:**
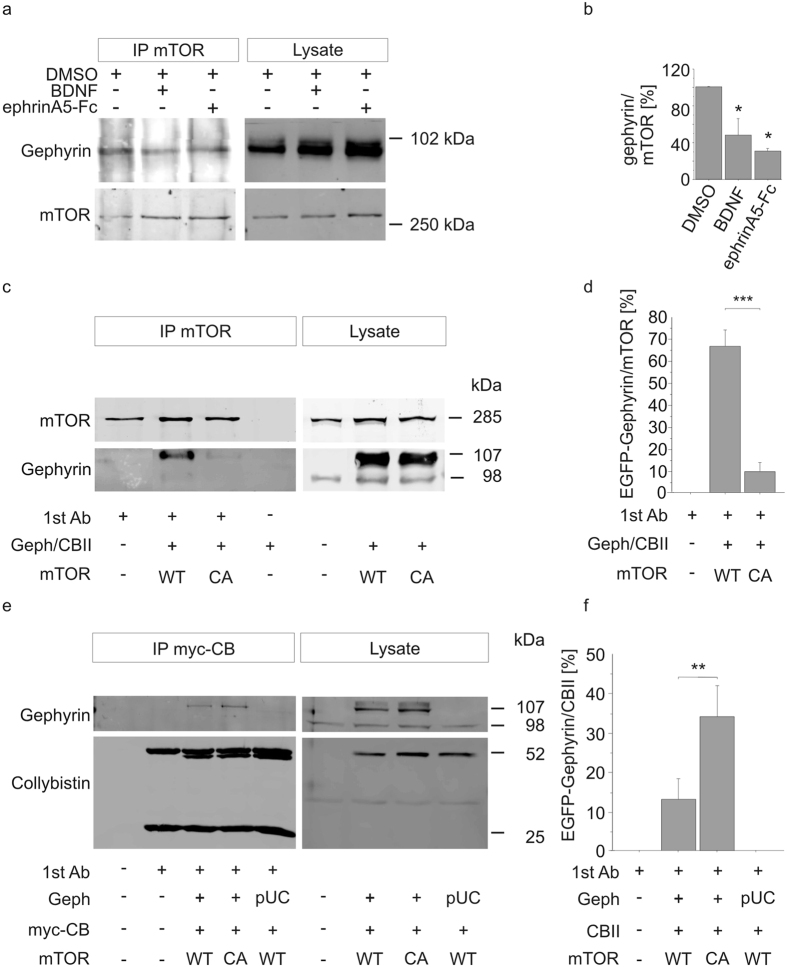
Differential interactions of activated mTOR with gephyrin and collybistin. (**a,b**) Cortical neurons were treated with ephrin A5 or BDNF for mTOR stimulation. Cell lysates were prepared for subsequent immunoprecipitation of endogenous mTOR and analysis of coprecipitated gephyrin after western blotting. Decreased mTOR-gephyrin interactions were observed after mTOR stimulation. (**b**) Densitometric quantification of bands depicted in (**a**) and determination of the gephyrin/mTOR ratio; n = 3, ANOVA, Fisher-PLSD; error bars, S.E.M. (**c–f**) Expression of ca-mTOR decreased EGFP-gephyrin/mTOR and increased collybistin/mTOR interactions. HeLa cells were transfected with expression vectors for wild type (WT) or constitutively active (CA) mTOR, EGFP-gephyrin (Geph) and myc-tagged collybistin (myc-CB). In some samples plasmid pUC18 (pUC) was added for equal amounts of transfected DNA. EGFP-gephyrin levels were related to the corresponding mTOR or collybistin signals. mTOR-gephyrin coprecipitation; n = 3; ***p < 0.0001. mTOR-myc-collybistin; n = 4; **p < 0.01; Kruskal-Wallis-Test, followed by Tukey-Test; error bars, S.E.M.

mTOR was previously shown to interact with gephyrin^9^,^29^,^30^. Therefore, we addressed the impact of mTOR activation on mTOR-gephyrin interactions. Primary neurons were exposed to ephrin A5-Fc or BDNF (positive control^9^) prior to immunoprecipitation of mTOR and analysis of coprecipitating gephyrin. In comparison to the control (100%), co-precipitation of gephyrin was decreased by both ephrin A5 (30%) and BDNF (48%) stimulation (Fig. 8a,b). We next examined whether mTOR activation by itself is sufficient for the release of mTOR-gephyrin interactions. HeLa cell lysates were prepared for immunoprecipitation after transfection with ca-mTOR or wt-mTOR along with expression plasmids for EGFP-gephyrin and myc-tagged collybistin (Fig. 8c–f). Western blot analysis of input lysates confirmed expression of mTOR, EGFP-gephyrin and myc-collybistin. In addition to EGFP-gephyrin (107 kDa), endogenous gephyrin of 98 kDa molecular mass was detected (Fig. 8c). Western Blotting revealed that EGFP-gephyrin coprecipitates with wt-mTOR as shown previously^9^,^29^. In comparison to cells expressing wt-mTOR (67%), the amount of co-precipitating EGFP-gephyrin was significantly reduced in the presence of ca-mTOR (9%, p < 0.0001) while no EGFP-gephyrin bands were observed in the absence of EGFP-gephyrin/collybistin or without the precipitating mTOR antibody.

Interactions of gephyrin with collybistin for membrane translocation represent a crucial mechanism for the stabilisation of GABAergic synapses^6^. To investigate this interaction in the context of mTOR activation, myc-tagged collybistin was immunoprecipitated from HeLa cell lysates and coprecipitated EGFP-gephyrin was analyzed by western blot (Fig. 8e,f). In the presence of ca-mTOR, significantly more EGFP-gephyrin (34%) coprecipitated with myc-collybistin (p < 0.01) than after transfection of wt-mTOR (13%). Hence, activation of mTOR is linked to a reduction of mTOR-gephyrin interactions, which coincides with enhanced association with collybistin. In conclusion, mTOR stimulation released the gephyrin-mTOR complex and allowed for enhanced interaction with collybistin.

## Discussion

Here we have shown that the receptor tyrosine kinase EphA7 is required for the stabilisation of basket cell innervation specifically on proximal dendrites and somata of dentate gyrus granule cells. *In vivo* electrophysiology and behavioural tests revealed a requirement of EphA7 expression for LTP and hippocampus-dependent learning. *In vitro* experiments indicated that EphA7-dependent gephyrin clustering relies on the protein kinase activity of EphA7 and depends on the activation of mTOR. mTOR activation coincides with reduced gephyrin-mTOR interaction and increases interaction of gephyrin with collybistin.

Interneurons forming GABAergic synapses in the CNS comprise different classes, which can be distinguished by anatomical, physiological and biochemical parameters^1^. Different compartments of principal neurons are addressed by specific interneurons that contribute to the control of principal neuron activity by spatially and temporally regulated modulation^31^. Here we show that EphA7 is specifically involved in stabilisation of GABAergic synapses of PV-positive basket neurons on proximal dendrites and somata of adult neurons. Likewise, EphA7 was suggested to be implicated in the developmental maturation of excitatory synapses^22^. The failure to detect EphA7 functions for the stabilisation of inhibitory synapses so far might rely on the use of electrophysiological measurements which are difficult to perform with cultured neurons *in vitro* that contain only few interneurons. Control of inhibitory input at specific subcellular compartments was also observed for the cell adhesion molecule neurofascin, which is specifically required for the stability of axo-axonic synapses formed by chandelier cells, another type of parvalbumin-positive interneurons^32^. Neurofascin is specifically expressed on axon initial segments and controls gephyrin clustering via FGFR1 activation. It is of note that EphA7 is involved in the stabilisation of synapses formed by parvalbumin-positive basket cells, while FGFR1 accounts for the stabilisation of synaptic terminals of parvalbumin-positive chandelier cells^32^. Thus, stabilisation of synapses formed by two distinct classes of parvalbumin-positive interneurons is provided by two different receptor tyrosine kinases. Receptor tyrosine kinase trkB might also be involved in the compartmental control of inhibitory input. Indirect evidence suggested stabilisation of perisomatic synapses of CA1 pyramidal neurons after activity-dependent upregulation of BDNF^33^. Since EphA and trkB signalling converge on common signalling pathways^34^,^35^, innervation of basket cells may be controlled by the combined action of EphA7 and trkB either by direct or indirect interactions of both receptors.

Our results show that proper GABAergic connectivity dependent on EphA7 can be considered as a critical determinant for LTP of granule cells and for their ability to contribute to hippocampus-dependent tasks. Decreased LTP after EphA7 knockdown may be explained by an impact on decreased basket cell connectivity. Basket cells are strategically positioned to control spiking and synchronous activity^36^. Eventually, LTP is impaired due to improper timing of backpropagating axon potentials after depletion of perisomatic synapses. However, further investigations are required for a final interpretation due to the high complexity of the local circuitry with its connections among the same and different neuronal populations^37^. In addition to long-term alterations in synaptic plasticity of principal cells in the dentate gyrus, we also examined short-term alterations in local circuit activity. Dentate gyrus local circuit activity and plasticity was assessed by the application of three different protocols that reflect inhibitory GABAergic modulation of granular cells. Results reveal that EphA7 down regulation did not affect inhibitory activity as assessed by these protocols.

In addition to LTP impairment, we found impaired learning under stress following downregulation of EphA7. EphA7 knock down rats showed less improvement in the two way shuttle avoidance task which may indicate impaired ability to cope with stressful situations. Recently, it was suggested that the dentate gyrus is associated with affective regulation and particularly is involved in stress processing^38^. Our results reveal that very specific and local modification of GABAergic input in the dentate gyrus are sufficient to induce significant behavioral alterations and support the role GABAergic modulation in stress processing^39^. It is of note that the assignment of LTP and hippocampal learning to the functionality of basket cells contrasts with the function of parvalbumin-positive chandelier cells. Interference with chandelier cell input at the axon initial segment of granule cells in the dentate gyrus did not impact LTP and memory functions. Instead, feedback inhibition became impaired along with increased fear levels^18^. Thus, two classes of parvalbumin-positive interneurons, namely chandelier- and basket cells, play specific roles in the control of principal neuron activity.

In neurodevelopment, EphA7 exerts repulsive functions for outgrowing axons, while in our system a completely different function in the stabilisation of inhibitory synapses was shown. Actually, these different functions could be explained by the selective activation of EphA7 downstream signalling pathways. For axon guidance and reorganization of the cytoskeleton, EphA receptors interact with the guanine exchange factor ephexin and thereby activate GTPase rhoA and repress Rac1 and cdc42^40^. Our results link EphA activation to the PI3K-Akt-mTOR pathway to be seen in the background of contrasting reports demonstrating the versatile nature of Eph-dependent signalling cascades: (1) PI3K activity can be induced or suppressed by EphA2 upon ephrin A1 stimulation, dependent on the cell type^41^,^42^; (2) EphA signalling is able to suppress mTOR activity^43^, while our experiments indicate that ephrin A5 induced mTOR; (3) EphA7 protein kinase activity is required for gephyrin clustering as shown here, whereas activity-dependent plasticity related to EphA4 is independent of the protein kinase activity indicating distinct signalling pathways for the control of excitatory and inhibitory plasticity^24^,^44^. In conclusion, our results indicated a link of EphA7 signalling to mTOR activation and concomitant control of gephyrin clustering although the precise connection between EphA7 activation and the induction of PI3K-mTOR signalling remains to be elucidated. Furthermore, we cannot formally exclude a contribution of further mTOR mechanisms to the control of inhibitory synapse stabilisation. However, mTOR-dependent control of translation appeared to be less probable since neither gephyrin nor collybistin protein expression were increased after expression of ca-mTOR or induction by ephrin A5-Fc.

In summary, our results imply a function of EphA7 for the stabilisation of basket cell terminals at granule cells of the dentate gyrus. While the precise link between EphA7 stimulation at the membrane and the induction of gephyrin clustering remains open, it can be concluded that the PI3K-Akt-mTOR pathway serves as common downstream target of trkB and EphA7 induced gephyrin clustering.

## Materials and Methods

### Lentiviral transduction

Lentiviral vector pLenti/CEW was described previously^32^. shRNAs either serving as a control (shCTR) or for the knockdown of EphA7 (sh1737, sh392) were transferred into pLenti/CEW via Gateway^®^-recombination (Life Technologies GmbH, Darmstadt, Germany) and lentiviral particles were produced according to the manufacturer’s protocols (viral titers approx. 5 × 107 TU/ml) (Virapower™ Lentiviral Expression System, Life Technologies GmbH, Darmstadt, Germany).

### Animals, stereotactic surgery, transcardial perfusion

For histological analyses, adult female Sprague Dawley rats (250 g at time point of surgery) were supplied by Janvier Labs, Saint-Berthevin Cedex, France. Animals were housed, cared for and used during surgical procedures in accordance with the European Union recommendations for the care and use of laboratory animals (2010/63/EU) and as approved by the regional authority (Regierungspräsidium Tübingen). For behavior and *in vivo* electrophysiology, male Sprague Dawley rats were supplied by Harlan Laboratories, Jerusalem. All procedures were carried out according to the NIH guide for the care and use of laboratory animals and as approved by the Haifa University Animal Care Committee. Adequate measures were taken to minimize pain and discomfort for the animals.

Animals were deeply anesthetized by injection of Ketamine/Xylazine (100 mg/kg and 10 mg/kg, respectively). Stereotaxic injections of lentiviral suspensions into the dorsal dentate gyrus (AP: −2.9 mm, ML: +/−2.5 mm, DV: −4.3 mm; all coordinates relative to Bregma) were performed using a Lab Standard^TM^ Stereotaxic Instrument (Stoelting Co., Wood Dale, USA) connected to a 701 RN Hamilton syringe (10 μl, 30 gauge, pst 4; CS-Chromatographie Service GmbH, Langerwehe, Germany). For fixation of brain tissue, animals were deeply anesthetized as described above and perfused transcardially with 200 ml 1 × PBS followed by 500 ml 4% paraformaldehyde/PBS.

### Cell culture and transfection

Culture and transient transfection of HeLa cells and primary neurons were performed as described previously^9^. Primary hippocampal neurons were cultured at high density (2 × 10^5^ cells/cm^2^). Transfection was performed at 10 days *in vitro* (DIV) and cultures were processed for imaging at 17 DIV. Equal amounts of plasmid DNA were transfected in all cases.

### Immunohistochemistry, immunocytochemistry

Perfusion-fixed brains were washed in PBS and cut into 100 μm slices using a vibrating microtome (Vibratome VT1000S, Leica Microsystems, Wetzlar, Germany). After permeabilization (0.6% Triton X-100), slices were blocked by 1 × BMB blocking reagent/PBS (Roche, Mannheim, Germany) for 1 h at room temperature. Further processing of brain slices and immunocytochemical staining was performed as described previously^32^. Specimens were mounted on microscopic slides using Dako Fluorescent Mounting medium (Dako GmbH, Hamburg, Germany). HeLa cells or cultured neurons were fixed with 4% of paraformaldehyde/PBS for 15 minutes and permeabilized prior to immunostaining. Confocal fluorescence images were acquired using a Zeiss LSM510 Meta confocal microscope equipped with a 63 × Plan-Apochromat oil immersion objective (NA 1.4; Carl Zeiss AG, Oberkochen, Germany). Images were analyze using IMARIS (Bitplane, Zurich, Switzerland).

### Immunoprecipitation and western blotting

For immunoprecipitation, cell lysates were prepared from primary cortical neurons or HeLa cells and submitted to immunoprecipitation. Samples of the lysates (input) or of the immunoprecipitate were seprarated by SDS PAGE followed by western blotting and detection by the ECL system (GE Healthcare Europe, GmbH, Freiburg, Germany). Alternatively, proteins were detected by fluorescence labeled secondary antibodies and a Typhoon Trio Imager (GE Healthcare Europe, GmbH, Freiburg, Germany). Densitometric analysis was performed using the gel analyzer function of the ImageJ software (Wayne Rasband, NIH, Bethesda, MD).

### Statistical analysis

Each experiment was performed at least three times. The number of experiments and different statistic evaluations (Statview software) including ANOVA-Fisher PLSD, Kruskal-Wallis followed by a Tukey test are indicated in the respective figure legends. Bar charts depict data with standard error of the mean.

See supplemental experimental procedures online for comprehensive descriptions of analytical methods used for this study.

## Additional Information

**How to cite this article**: Beuter, S. *et al*. Receptor tyrosine kinase EphA7 is required for interneuron connectivity at specific subcellular compartments of granule cells. *Sci. Rep.* **6**, 29710; doi: 10.1038/srep29710 (2016).

## Supplementary Material

Supplementary Information
