# Supplementary material for: Receptor tyrosine kinase EphA7 is required for interneuron connectivity at specific subcellular compartments of granule cells
Source: Sci Rep. 2016 Jul 13;6:29710. doi: 10.1038/srep29710 (PMC4942821; doi:10.1038/srep29710)
Supplement: Supplementary Information [file srep29710-s1.pdf]

Receptor tyrosine kinase EphA7 is required for interneuron connectivity at specific subcellular compartments of granule cells

Simone Beuter, Ziv Ardi, Omer Horovitz, Jennifer Wuchter, Stefanie Keller, Rinki Saha, Kuldeep Tripathi, Rachel Anunu, Orli Kehat, Martin Kriebel, Gal Richter-Levin and Hansjürgen Volkmer

Supplementary figures and methods

Figure S1: Expression of EphA7 *in vivo* and *in vitro*.
Figure S2: Knockdown of EphA7 at the protein and mRNA level.
Figure S3: Overexpression of wild type EphA7 does not increase gephyrin cluster size
Figure S4: S6K is activated by ephrin A5-Fc stimulation
Supplemental experimental procedures

Figure S1

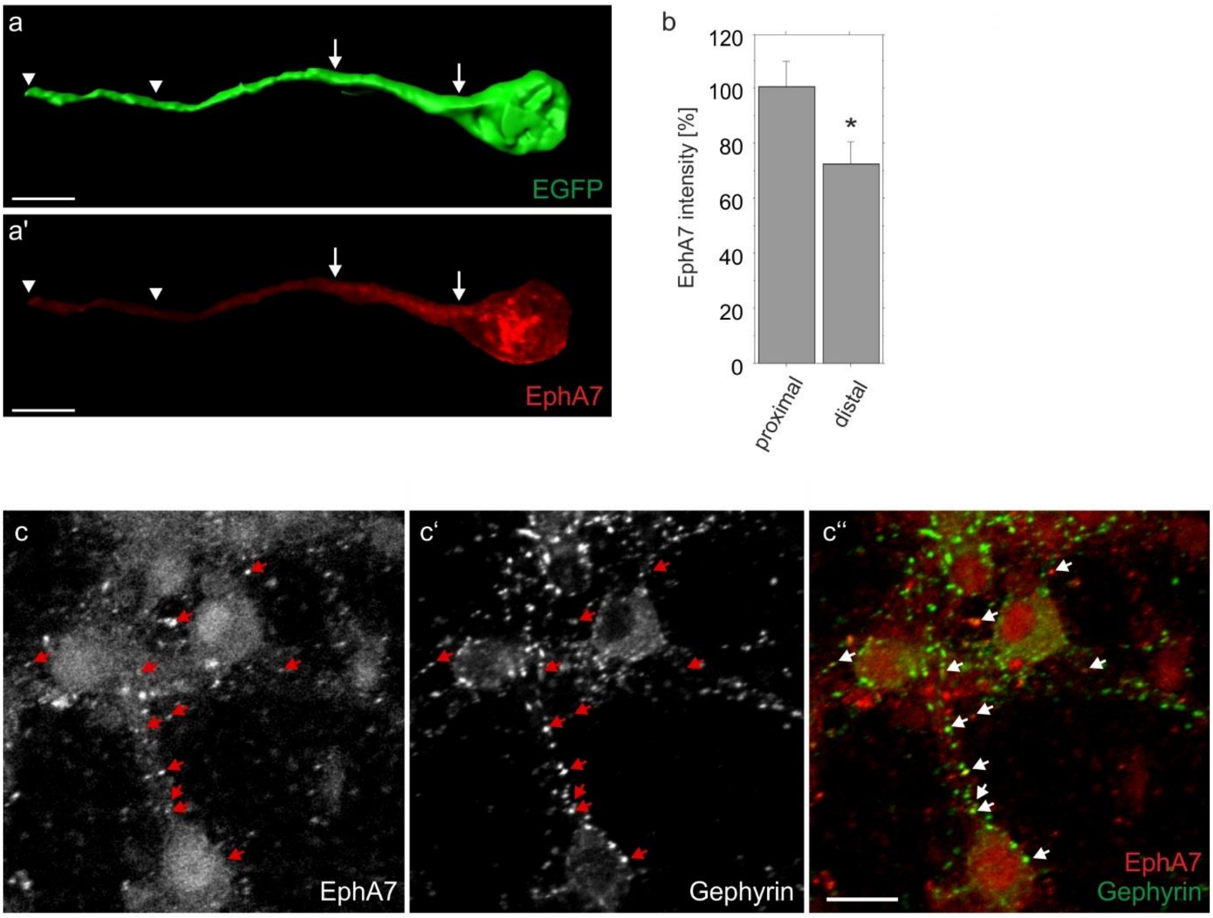

EphA7 expression *in vivo* and *in vitro*. For labeling of granular cells, the principal neurons of the dentate gyrus, animals were injected with a lentiviral vector expressing EGFP under the control of the CamKII promoter. Prominent EphA7 expression (a’) was observed on somata and proximal dendrites of EGFP-positive granular neurons (a). Scale bars: 10 µm. For the analysis of the subcellular distribution of EphA7, fluorescence voxel intensities of 20 µm segments of proximal (arrows) and distal (arrow heads) parts of dendrites within EGFP surface masks in a were quantified in b (n=12; *p<0.05; ANOVA, Fisher-PLSD; error bars: S.E.M.). Distal dendritic segments showed strongly reduced labelling (p<0.05). (c-c’’) Dissociated hippocampal neurons were stained for EphA7 (c) and gephyrin (c’), a marker of GABAergic postsynapses. Gephyrin clusters colocalizing with EphA7 immunoreactivity (red

arrowheads in c and c’, white arrowheads in c’’) were identified on EphA7-positive somata and neurites. Scale bars: 10 µm (a), 20 µm (c).

Figure S2

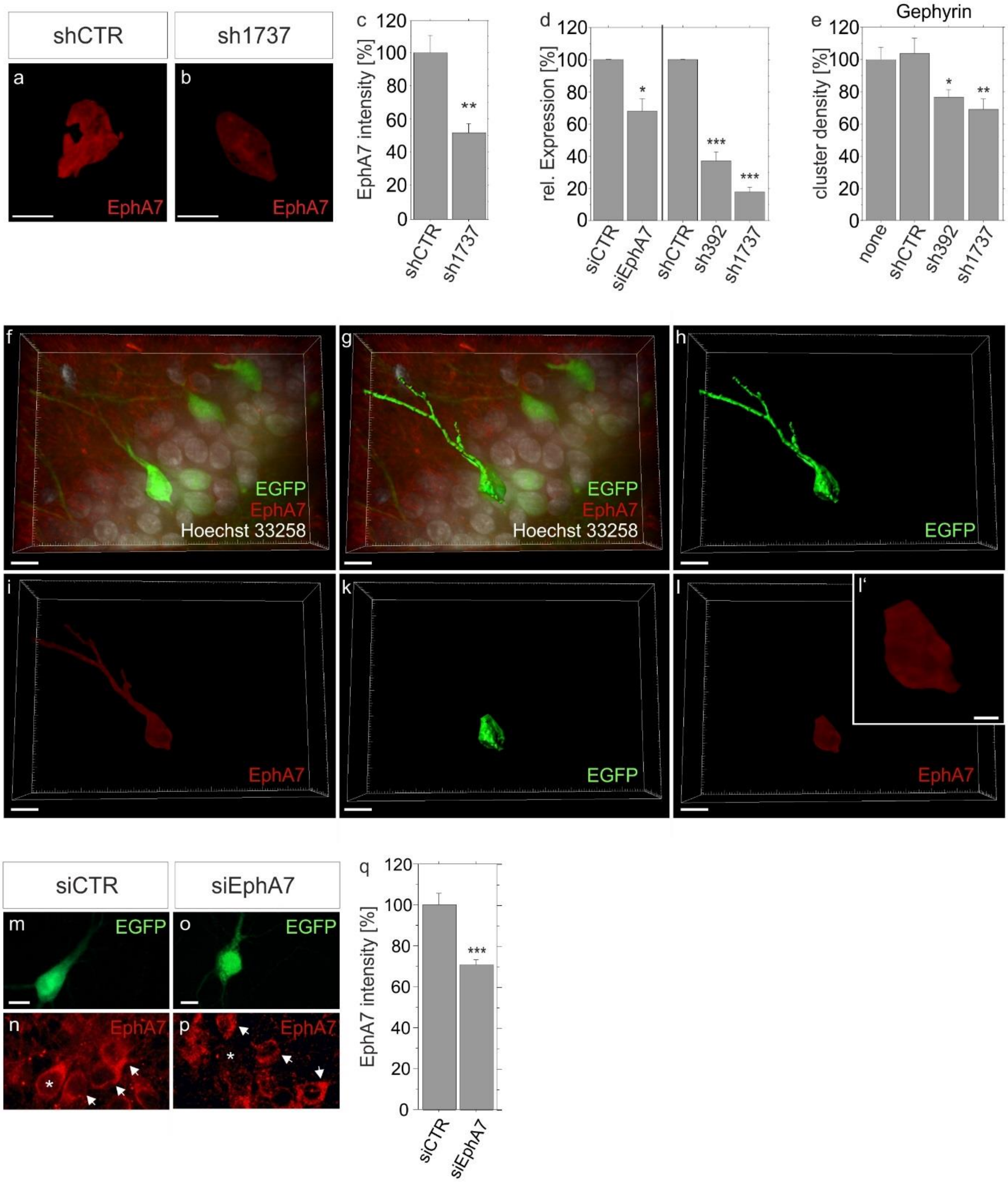

Knockdown of EphA7 at the protein and mRNA level. shRNA sequences were bioinformatically tested for off-target recognition. No other mRNAs including unrelated Eph receptors are expected to be targeted by EphA7 knockdown sequences. (a,b) Representative images of granule cell somata after transduction of granule cells with control or EphA7

knockdown lentivirus and immunostaining for EphA7 expression. Scale bars: 10 µm. (c) Quantification of (a,b) representing mean voxel intensities of EphA7 immunoreactivity in cell somata. n=25; **p<0.01; ANOVA, Fisher-PLSD. (d) qRT-PCR for the quantification of knockdown efficiencies of siRNA and lentiviral shRNA vectors. Hippocampal neurons were transduced with two different lentiviral vectors, sh392 and sh1737, targeting independent sequences of EphA7 mRNA, or nucleofected with an siRNA specific for EphA7 (siEphA7). siCTR, shCTR represent ineffective controls. Data were normalized to GAPDH mRNA levels. n=3; *p<0.05, ***p<0.0001. (e) Lentiviral shRNA vectors both reduced gephyrin cluster density in hippocampal neurons. n=40; *p<0.05; **p<0.01. ANOVA, Fisher-PLSD; error bars: S.E.M. (f-l) Illustrated workflow using Imaris software to yield regions of interest representing granule cell somata and allowing for image based quantification of EphA7 immunoreactivity *in vivo* after lentiviral transduction of granule cells (EGFP) as shown in panels a-c. Scale bars: 10 µm (f-l), 4 µm (l'). (f) Confocal stack showing EGFP-positive infected granular cells as well as EphA7 staining. (g) Segmentation of one cell to create an EGFP mask isolated in (h). (i) Dendritic and somatic EphA7 staining as defined by colocalizing EphA7-positive voxels within the EGFP mask shown in (h). (k) Extraction of the soma based on EGFP staining. (l) Representation of EphA7 staining co-localizing with the EGFP mask shown in (k). (m-p) Hippocampal rat neurons transfected with siRNAs as indicated and immunocytochemically stained against EphA7 three days after transfection. Cotransfection of an EGFP expressing plasmid (pEGFP-N1) served to visualize successfully transfected neurons. Asterisks in (n) and (p) mark transfected neurons as identified in (m,o), arrows point at untransfected cells showing unaffected EphA7 expression. (q) Quantification of EphA7 immunoreactivity on somata of siRNA transfected neurons as shown in (m-p) and normalized to siCTR. ANOVA Fisher-PLSD, n=35; siCTR vs. siEphA7: p<0.001; error bars: S.E.M.

Figure S3

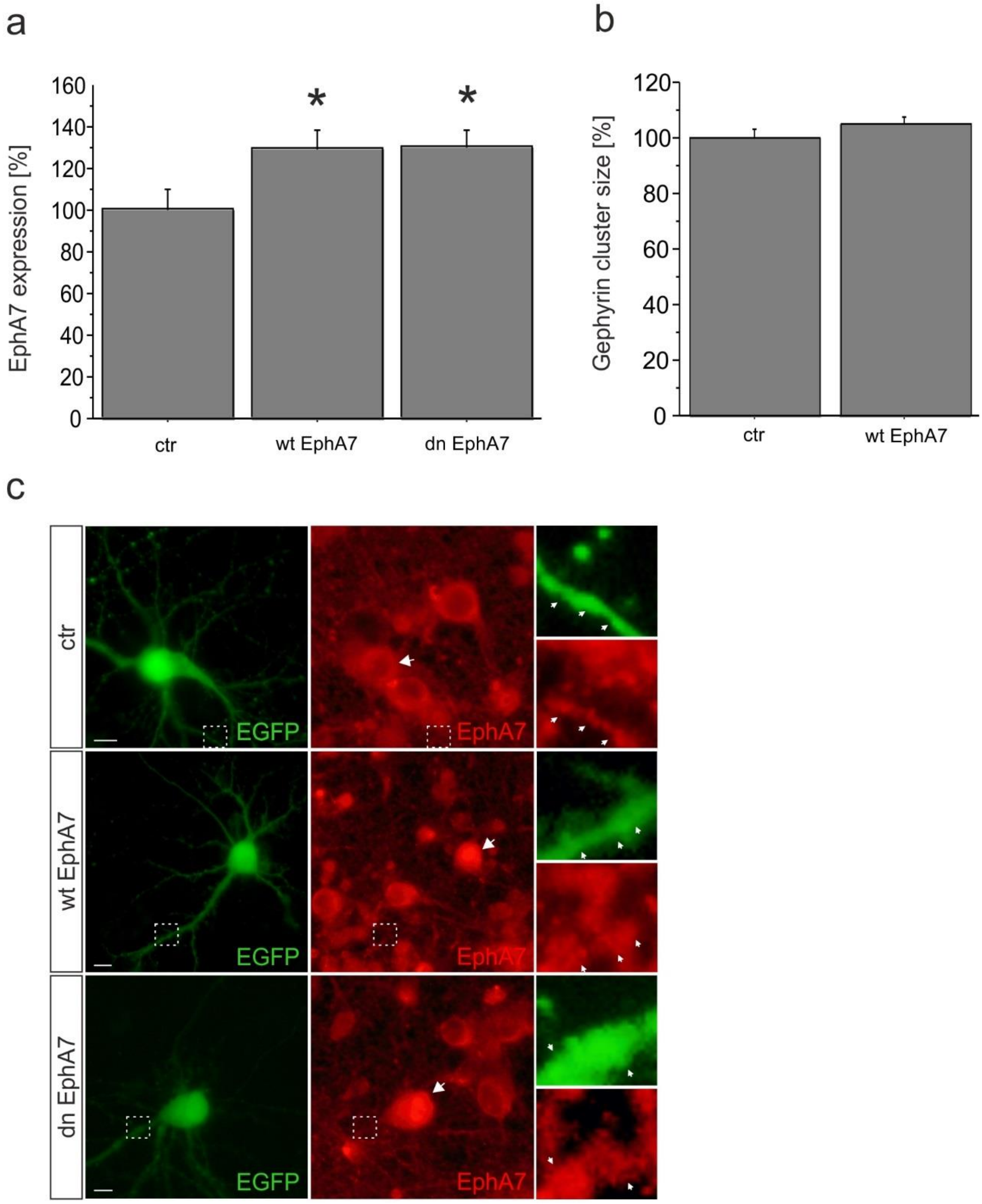

Overexpression of EphA7 does not increase gephyrin cluster size. (a) Hippocampal neurons were transfected with control, wild type EphA7 (wt EphA7) or dominant negative EphA7 (dn EphA7) expression plasmids. EphA7 expression was examined by quantitative immunofluorescence analysis six days after transfection (ANOVA Fisher-PLSD, n>20; ctr vs.

wt EphA7: p=0.03; ctr vs. dn EphA7: p=0.02. error bars: S.E.M.) (b) After immunocytochemical staining for gephyrin, volumes of individual gephyrin clusters on proximal dendritic segments were determined and mean cluster volumes were compared between experimental groups (n=1124 clusters for control, n=1396 clusters for wild type EphA7; p=0.1998, ANOVA, Fisher-PLSD; error bars: S.E.M.). Samples analysed are identical to those in figure 4. (c) Immunocytochemical EphA7 staining of transfected hippocampal rat neurons quantified in (a). To visualize successfully transfected neurons, an EGFP expressing plasmid (pEGFP-N1) was cotransfected. Large arrows indicate EGFP-positive neuronal somata with increased EphA7 immunoreactivity after transfection of either wt EphA7 or dn EphA7. Insets depicted by squares in corresponding images showing EGFP or EphA7 staining, respectively, were enlarged in the right-hand column. Small arrows indicate dendritic processes of transfected cells expressing EphA7. Scale bars: 10µm.

Figure S4

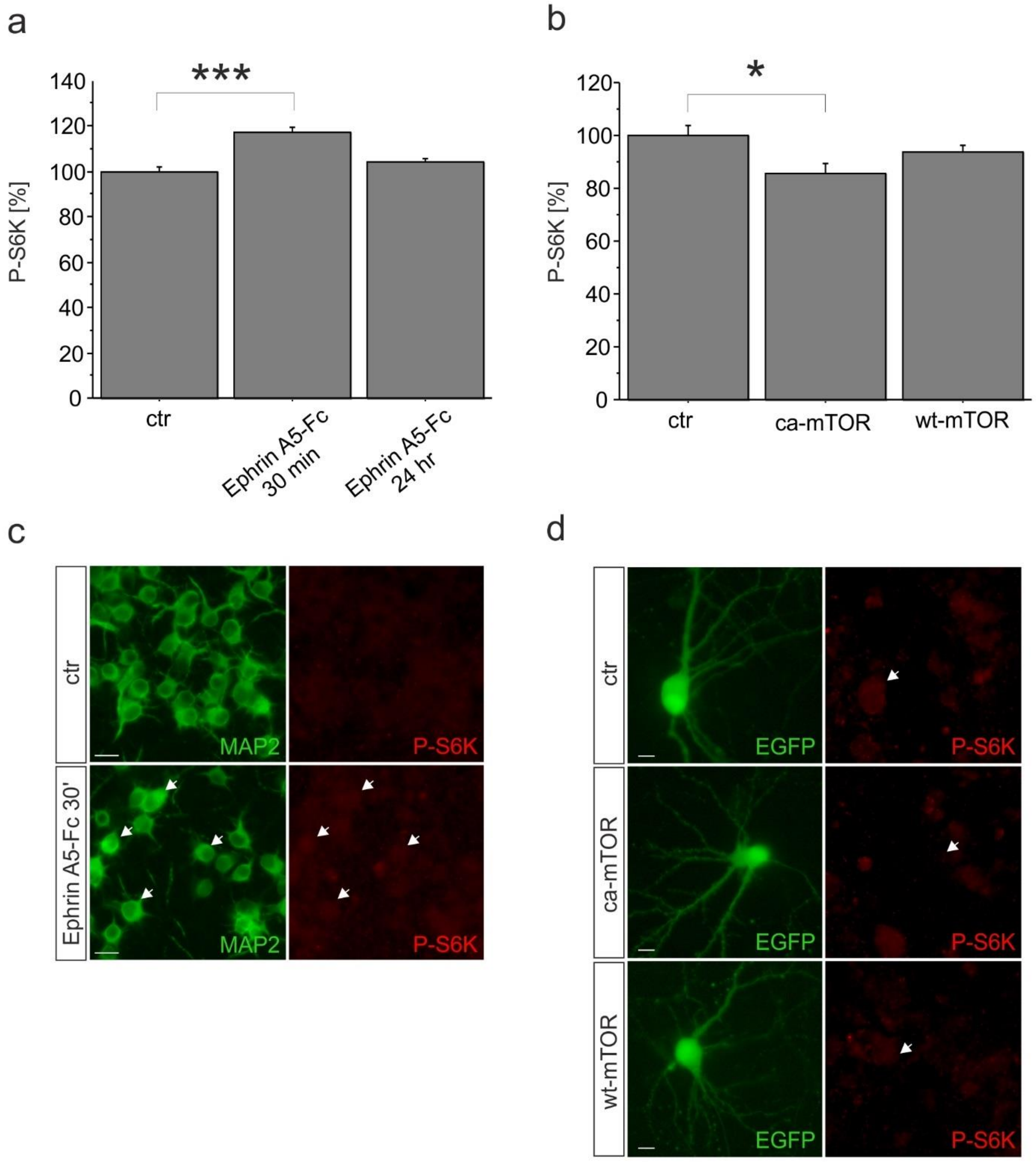

S6K is activated by ephrin A5-Fc stimulation. (a) Neurons were stimulated with ephrin A5-Fc for 30 min or 24 hrs, respectively. Thr389 phosphorylation of S6K was examined by quantitative immunofluorescence analysis (ANOVA Fisher-PLSD, n=50; ***: p<0.0001 as compared to the control (ctr), error bars: S.E.M.). (b) After transfection with control plasmids

(ctr), ca-mTOR or wt-mTOR expression plasmids, neurons were further cultivated for 7 days and stained for S6K phosphorylation (ANOVA Fisher-PLSD, n>25; p=0.0035 for ca-mTOR and p=0.1747 for wt-mTOR as compared to the control (ctr), error bars: S.E.M.). (c) Cultured rat neurons treated as indicated and immunocytochemically stained against microtubule-associated protein 2 (MAP2) and S6K phosphorylated at Thr389 (P-S6K). Arrows indicate neuronal somata with increased P-S6K immunoreactivity after treatment with ephrin A5-Fc for 30 minutes. Scale bars: 20µm. (d) Cultured rat neurons transfected as indicated and immunocytochemically stained six days after transfection against S6K phosphorylated at Thr389 (P-S6K). To visualize successfully transfected neurons, an EGFP expressing plasmid (pEGFP-N1) was cotransfected. Arrows indicate somata of transfected neurons. In the case of ca-mTOR transfection, a reduction in P-S6K immunoreactivity is observed. Scale bars: 10µm.

Supplemental experimental procedures

Plasmids, siRNAs

mTOR expression plasmids pcDNA3-Au1-mTOR-wild type and pcDNA3-AU1-mTOR-S2215Y were obtained from Addgene (http://www.addgene.org, Cambridge, UK; plasmids 26036, 26037; (Sato et al., 2010)). Plasmids pEGFP-C2/gephyrin (kindly provided by H. Betz and G. O'Sullivan, Frankfurt, Germany) and pRK5myc/collybistin SH3- (a gift from R. J. Harvey, London, UK) were used for EGFP-gephyrin or collybistin expression, respectively. Further plasmids used were pEGFP-N1 (Clontech Laboratories Inc., Mountain View, USA), and pCMV6_XL4/EphA7 (Origene, Rockville, USA). A kinase-deficient mutant (K665M) of human Eph receptor A7 (Varjosalo et al., 2008) was created with QuickChange® Lightning site directed Mutagenesis Kit (Agilent Technologies, Böblingen, Germany) using the following primer set: EphA7dn-F: (GAGATGTTGCAGTAGCCATAATGACCCTGAAAGTTGGTTACAC) and EphA7dn-R: (GTGTAACCAACTTTCAGGGTCATTATGGCTACTGCAACATCTC). siRNAs targeting rat mRNAs were supplied by Life Technologies GmbH, Darmstadt, Germany: siCTR (siRNA AM4621); siEPHA7 (siRNA ID s139899); siFGFR (siRNA ID s5164); siACVR2A (siRNA ID s131048).

Transfection of HeLa cells

Cultivation and transfection of HeLa cells was performed essentially as described previously (Kriebel et al., 2011). HeLa cells were cultivated at a density of 1 x 10^5 cells/cm^2 in 24 well plates or at a density of 1.75 x 10^5 cells/cm^2 in 6 well plates. For transfection, up to 0.6 µg of

pcDNA3-AU1-mTOR-wild type (wt-mTOR) or pcDNA3-AU1-mTOR-S2215Y (ca-mTOR), pEGFP-C2/gephyrin and pRK5myc/collybistin SH3- were diluted in 50 µl (24 well) or 250 µl (6 well) of OptiMEM (Life Technologies GmbH, Darmstadt, Germany). Subsequently, 2 µl (24 well) or 5 µl (6 well) of Lipofectamine 2000 (Life Technologies GmbH, Darmstadt, Germany) were diluted in a total volume of 50 µl (24 well) or 250 µl (6 well) of OptiMEM. After incubation of 20 min, DNA and Lipofectamine solutions were combined and added to the cells. The cells were fixed for immunostaining or harvested for immunoprecipitation 24 hrs after transient transfection.

Neuronal cell culture, transfection and lentiviral transduction

Primary hippocampal or cortical neurons were prepared from E17 rat embryos of either sex as described previously (Kriebel et al., 2011) and cultivated in NMEM/B27 medium at a density of 1.8 x 10^5 cells/cm^2 for 96-well plates, or of 2 x 10^5 cells/cm^2 for 6- or 12- well plates, respectively. Hippocampal neurons were transiently transfected with 500 ng of plasmid DNA or siRNA (final concentration, 26.6 nM) using 1 µl of Lipofectamine 2000 (Life technologies GmbH, Darmstadt, Germany) in 80 µl of plating medium at DIV 10. For lentiviral transduction, hippocampal neurons were cultured in 96-well plates and infected with a total volume of 2.5 µl lentiviral suspension at DIV 2. Inhibitors and recombinant proteins were dissolved in water, PBS or dimethylsulfoxide (DMSO) and added to hippocampal neurons at DIV 16 to final concentrations of 10 µM for MEK2 inhibitor 1,4-diamino-2,3-dicyano-1,4-bis(2-aminophenylthio) butadiene (U0126) (Merck KGaA, Darmstadt, Germany), 10 µM for PI3K inhibitor 2-morpholin-4-yl-8-phenylchromen-4-one (LY294002) (Merck KGaA, Darmstadt, Germany), 200 nM for mTOR inhibitor rapamycin (Merck KGaA, Darmstadt, Germany) and 10 ng/ml for trkB ligand BDNF/H_2O (New England Biolabs GmbH, Frankfurt, Germany). 20 µg/ml ephrin A5-Fc/PBS (R&D Systems, Minneapolis, USA) or NF166-Fc/PBS (Pruss et al.,

2006) were preclustered with 10 µg/ml anti-human IgG (Dianova GmbH, Hamburg, Germany) for 30 min and subsequently applied to the culture media at 100 ng/ml. For stimulation experiments, cells were preincubated with inhibitors or DMSO 30 min before application of BDNF, ephrin A5-Fc or NF166-Fc. Analyses were performed 24 h after addition of the reagents.

For quantification of EphA7 and GAPDH mRNA expression via qRT-PCR, the following assays were used: GAPDH (VIC/MGB), Rn01775763_g1; EphA7 (FAM/MGB), Rn00592517_m1 (Life Technologies GmbH, Darmstadt, Germany).

Immunocytochemistry

For immunocytochemical analyses, the following antibodies were used: mouse monoclonal anti-gephyrin (mAb7a; 1:100; Synaptic Systems, Göttingen, Germany), rabbit polyclonal anti-GAD65 (1:1000, Chemicon International, Inc., USA), rabbit polyclonal anti-MAP2 (1:1000; Merck KGaA, Darmstadt, Germany), rabbit polyclonal anti-PSD-95 (1:200; New England Biolabs GmbH, Frankfurt, Germany), rabbit polyclonal anti-γ2-subunit of $GABA_A$ receptors (1:200; Synaptic Systems, Göttingen, Germany). 24 hrs after transient transfection of HeLa cells or at DIV 17 for primary hippocampal neurons, cells were fixed with 4% paraformaldehyde/PBS for 10 min. After blocking and permeabilizing for 30 min with 0.2% Triton X-100 in PBS containing 1xBMB blocking reagent (Roche, Hamburg, Germany), cells were incubated with primary antibody (1xBMB) at 4°C overnight. Subsequently, cells were washed three times using PBS before the secondary antibody (1:500 in 1xPBS; Cy3/Cy5-coupled goat anti-mouse or goat anti-rabbit; Dianova GmbH, Hamburg, Germany) was added for 2 hrs at room temperature. Nuclei were stained using Hoechst 33258 (1:1000 in PBS; Sigma-Aldrich, St. Louis, USA).

Immunohistochemistry

For immunohistochemistry the following primary antibodies diluted in 10 % Normal Goat Serum/PBS (Abcam, Cambridge, UK) were used: mouse monoclonal anti-gephyrin (mAb7a, 1:100, Synaptic Systems, Göttingen, Germany), mouse monoclonal anti-VGluT1 (1:1000, Synaptic Systems, Göttingen, Germany), rabbit polyclonal anti-VGAT (1:3000, Synaptic Systems, Göttingen, Germany), rabbit polyclonal anti-VGSC (1:100, Sigma-Aldrich, St. Louis, USA), rabbit polyclonal anti-PSD-95 (1:1000, Abcam, Cambridge, UK), rabbit polyclonal anti-γ2-subunit of $GABA_A$ receptors (1:500; Synaptic Systems, Göttingen, Germany), and polyclonal anti-Eph receptor A7 (ab136095, 1:50, Abcam, Cambridge, UK). Perfusion-fixed brains were washed in PBS and cut into 50-70 μm slices using a vibrating microtome (Leica Mikrosysteme Vertrieb GmbH, Wetzlar, Germany). For staining of VGluT, VGAT, VGSC, PSD95, $GABA_A$ γ2, and EphA7, an antigen retrieval procedure was performed. Brain slices were incubated for 30 min at 80°C in sodium citrate at pH 8.5 (Jiao et al., 1999). Slices were permeabilized (0.6% Triton X-100 in PBS) and blocked with 10 % Normal Goat Serum/PBS (Abcam, Cambridge, UK) for 1 h at room temperature. The brain slices were incubated overnight at room temperature in primary antibody diluted in blocking solution and then washed three times for 15 min using PBS before the secondary antibody (1:500 in 10 % Normal Goat Serum/PBS; Cy3/Cy5-coupled goat anti-mouse, goat anti-rabbit; Dianova GmbH, Hamburg, Germany) was added for 4 hrs at room temperature. After additional washing steps and nuclei staining with Hoechst 33258 (1:1000 in PBS; Sigma-Aldrich, St. Louis, USA), specimens were mounted on microscopic slides using Dako Fluorescent Mounting medium (Dako GmbH, Hamburg, Germany).

Immunoprecipitation and Western blot

18 hrs before lysis, HeLa cells were starved in serum-free medium and finally stimulated for 30 min with medium containing FCS. 24 hrs after transfection, HeLa cells were washed in ice-cold PBS before lysis. 150 µl of IP-lysis buffer (10mM Tris-HCl, pH 7.5, 100 mM NaCl, 10 mM EDTA, 0.5% Triton X-100, 0.5% desoxycholate) per well were added to HeLa cells cultivated in 6-well plates before further incubation for 10 min on ice. Primary cortical neurons were cultivated in 12-well plates for 17 days. BDNF was added to the culture at a final concentration of 200 ng/ml, while preclustered ephrinA5-Fc was applied at 100 ng/ml, each for 30 min. For each treatment, 6-8 wells of a 12-well plate were used and pooled later on. Cells were washed using ice cold PBS, then 50 µl of IP-lysis buffer (10 mM Tris-HCl, pH 7.5, 100 mM NaCl, 10 mM EDTA, 0.5% Triton X-100, 0.5% desoxycholate) was added to each well and incubated for 10 min on ice.

Using a cell scraper, cell lysates were collected, pooled, and centrifuged for 10 min at 4°C at 16,000 x g. Protein concentrations were determined (BCA assay kit; Thermo Fisher Scientific Inc., Waltham, USA), and 700 µg of total protein was incubated with a 1:100 dilution of anti-mTOR antibody (no. 2983; New England Biolabs GmbH, Frankfurt, Germany) or anti-myc antibody (Sigma-Aldrich, St. Louis, USA) and incubated at 4°C for at least 6 h on a rotating device. Subsequently, 60 µl of resuspended protein A-Sepharose (50% v/v; Sigma-Aldrich, St. Louis, USA) was added and incubated overnight at 4°C on a rotating device. Beads were washed by five alternating steps of centrifugation and resuspension in lysis buffer at 4°C.

Samples of either lysates or immunoprecipitates were separated by SDS-PAGE prior to Western blotting and transfer to nitrocellulose membranes. For protein detection, anti-gephyrin (1:1000; #147111, 3B11; Synaptic Systems, Göttingen, Germany), anti-mTOR (1:1000; no. 2972; New England Biolabs, Frankfurt, Germany), or anti-myc antibodies (Sigma-Aldrich, St. Louis,

USA) were applied. Horseradish peroxidase-coupled goat anti-rabbit secondary antibodies (Dianova GmbH, Hamburg, Germany) were applied for the analysis of primary neurons. Detection was achieved after application of ECL substrate (GE Healthcare Europe GmbH, Freiburg, Germany). For HeLa cells, Cy3- or Cy5-coupled goat anti-mouse or goat anti-rabbit (Dianova GmbH, Hamburg, Germany) secondary antibodies (1:500) were used and signals were detected using a Typhoon Trio Variable Mode Imager (GE Healthcare Europe GmbH, Freiburg, Germany).

For analysis of phosphorylated signaling components, cortical neurons were plated on 6-well plates at a density of 2 x 10^5 cells/cm^2. At DIV17, cells were pretreated for 30 min using DMSO, MEK2 inhibitor U0126, PI3K inhibitor LY294002, or mTOR inhibitor rapamycin. Cells were treated with BDNF or preclustered ephrin A5-Fc for 30 min before lysis using 150 µl of IP-lysis buffer. Samples were processed for Western blotting as described above. Primary antibodies were purchased from New England Biolabs, Frankfurt, Germany: anti-mTOR antibody (1:200; no. 2972), anti-ERK 1/2 (1:2000; no. 9102), anti-pmTOR antibody (1:200; no. 5536), anti-pERK 1/2 (1:2000; no. 4370). Anti-actin was supplied by Sigma-Aldrich, St. Louis, USA) and anti-gephyrin (1:1000; #147111, 3B11) by Synaptic Systems, Göttingen, Germany. Cy3- or Cy5-coupled goat anti-mouse or goat anti-rabbit antibodies were applied (1:500; Dianova GmbH, Hamburg, Germany) and detected using a Typhoon Trio Variable Mode Imager (GE Healthcare Europe GmbH, Freiburg, Germany). Densitometric analysis of band intensities was performed using the gel analyzer function of ImageJ software (Wayne Rasband, National Institutes of Health, Bethesda, MD).

Image acquisition, image analysis, and statistics

Confocal fluorescence images were acquired using a Zeiss LSM510 Meta confocal microscope or a Cell Observer SD equipped with a 63 x Plan-Apochromat oil immersion objective (NA 1.4; Carl Zeiss Microscopy GmbH, Göttingen, Germany). Images of HeLa cells and Z-stacks of primary hippocampal neurons and brain tissue were recorded. Acquired images were further processed and analyzed using Imaris software (Bitplane AG, Zurich, Switzerland). A surface mask was built for MAP2, VGSC or EGFP channels. This mask was used to build a new channel including synaptic spots (for inhibitory or excitatory markers) that co-localized to the MAP2, VGSC or EGFP mask. Then, a region of interest was built encompassing the proximal 20 µm of dendritic segments or 20 µm segments identified at 80 µm distance from somata (defined as "distal segments"), whole somata or the axon initial segment. A mask representative for synaptic punctae was super-imposed on the respective areas of interest. Imaris software was applied to calculate volumes and densities of synaptic punctae in the areas of interest. In the case of somata, gephyrin cluster densities were first normalized to the surface area of individual somata. For EphA7 expression, mean voxel intensities of EphA7 signals detected on cell somata or on 20 µm segments of proximal and distal parts of dendrites within EGFP surface masks were measured. Hoechst staining was used to define masks for nuclei, which were subsequently excluded from the analysis. Similarly, images of HeLa cells were processed for the determination of the number and area of EGFP–gephyrin signals. Within each experiment, all settings for exposure times, contrast, brightness, resolution and threshold values were kept constant. Statistical analyses were performed using StatView or JMP ® 10 software (SAS Institute Inc., North Carolina, USA). P-values were assigned as follows: *: $p<0.05$; **: $p<0.01$; ***: $p<0.0001$. At least three independent biological replicates were used for each experiment.

Behavioral Assessments

Two weeks following injections, rats were tested in the open field, elevated plus maze and two way shuttle tasks (as described below). The open field and elevated plus maze tests were conducted on the same day, the two way shuttle task was conducted the following day.

The open field test was carried out according to methods described previously (Avital et al., 2006). Briefly, the open field test consists of a square Plexiglas box (50 × 50× 38 cm) positioned in a dimly red-lit ventilated sound-attenuated room. The walls are painted black, the floor is white and divided by 0.3 cm-wide black lines into 25 equal squares of 10 × 10 cm each. After 5 min habituation to the room rats were placed at the corner of the open field facing the wall and were allowed to explore the novel environment for 5 min while their behaviour was recorded and analyzed via EthoVision XT8 tracking system (Noldus, Wageningen, Netherlands).

The elevated plus maze test was carried out as described previously (Pellow et al., 1985). Briefly, the maze was placed 50 cm above the floor and consisted of two open arms and two closed arms (with 30 cm high Plexiglas walls and no roof), arranged in a way that similar arms are opposite to each other. After 5 min habituation to the room, each animal was placed in the center of the maze facing an open arm and was allowed to explore the arena freely for 5 min. Behaviour was recorded and analyzed via the EthoVision XT8 tracking system.

For the two way shuttle avoidance task, adult rats were conditioned in a two way shuttle avoidance box (Panlab, Harvard apparatus, Spain) placed in a dimly-lit, ventilated and sound-attenuated cupboard. The rectangular chamber (60 x 26 x 28 cm) was divided by an opaque partition with a small passage (10 x 8 cm) into two equal sized cubicles. Both compartments' metal grid floors are weight sensitive; micro-switches transmit information on the rat's location to a computer-controlled and automated data collection program managing the conditioned

stimulus (CS) and the unconditioned stimulus (US) presentations. The CS was a tone produced by loudspeakers located on the distal walls of the compartments and the US is an electric foot shock. Information was also recorded concerning the rats' responses, which can be avoidance (shuttle during the CS), escape (shuttle during the US) or no escape (no shuttle during the US). Two training sessions were performed on two consecutive days. The first session began with 10 minutes of habituation to the apparatus and the second session began with one minute of habituation. Sessions consisted of 75 trials; each starting with the delivery of the CS for 10 sec (75 db, 3000 Hz) immediately followed by the US (0.8mA foot shock, 10 sec maximum) with an inter-trial interval of 30 ± 7.5 sec.

In vivo electrophysiology

About 10 days after behavioral testing, *in vivo* electrophysiology was performed on all rats. Adult rats were anesthetized (40% urethane, 5% chloral hydrate in saline, max. 0.5 ml/100 g i.p.) and placed in a stereotaxic apparatus. Body temperature (maintained at 37°C ± 0.5°C with Homeothermic Blanket System, Harvard apparatus, Dover, MA, USA) and level of anaesthesia were monitored and adjusted if needed during the experiment.

A glass recording electrode (tip diameter 2-5 µm), filled with a 2 M of NaCl solution, was inserted into the dentate gyrus of the dorsal hippocampus [coordinates (adult/adolescent): 4mm/3.5mm posterior to bregma, 2.5 mm/1.9 mm lateral to the midline]. A bipolar 125 µm stimulating electrode was placed in the perforant path [coordinates: 8.0mm/7.0mm posterior to bregma, 4.0 mm/4.2 mm lateral to the midline]. Depth of the electrodes was adjusted to yield maximal response (excitatory postsynaptic potential) recorded in the dentate gyrus. An additional stimulating electrode was positioned in the contra-lateral dentate gyrus to activate the commissural pathway [coordinates: 4mm/5.5mm posterior to bregma, -2.5 mm/-1.9 mm

lateral to the midline, depth 3.5 mm]. Evoked responses were digitized (10 kHz) and analyzed using a Cambridge Electronic Design (Cambridge, UK) 1401 and its Spike2 software (version 4.24). Off-line measurements were made of the field EPSP slope and population spike amplitude.

After electrode insertion, recording was allowed to stabilize for 30 min. Input-output relations were then examined, using average of 5 successive responses at 0.1 Hz for increasing stimulus intensities. Baseline field potential responses were recorded with a stimulus intensity of 50 % of the intensity that evoked maximum spike amplitude (monopolar pulses, 100 μs duration). Baseline measurements were then taken for 30 min (at 0.1 Hz) followed by three local circuit activity protocols.

Local circuit activity and frequency dependent modulation: frequency dependent inhibition was determined as described previously (Rosenblum et al., 1999; Sloviter, 1991). Ten baseline pulses were delivered to the perforant path at 0.1 Hz, followed by 10 pulses delivered at 1 Hz. Inhibition index was measured by the average population spike amplitude of the 1 Hz stimulation divided by that of prior stimulation at 0.1 Hz.

Local circuit activity and paired-pulse inhibition: As described in earlier studies(Andersen et al., 1966; Richter-Levin and Segal, 1991; Sloviter, 1991), paired-pulse inhibition was measured by applying five pairs of two constant stimuli to the perforant path at inter-stimuli interval of 15 ms. The inhibition index was measured by the averaged population spike amplitude of the response to the second stimulus divided by that of the first stimulus.

Local circuit activity and commissural modulation: The DG commissural pathway was activated by stimulating the contralateral DG at intervals of 15 and 30 ms prior to perforant

path stimulation as described before (Richter-Levin and Segal, 1991; Yarom et al., 2008). This was repeated five times. The inhibition/excitation index was measured by the averaged population spike amplitude of the five second pulses divided by the average of the baseline measurement.

LTP induction

Following the application of local circuit activity protocols, some of the rats were also tested for LTP induction. Baseline measurements were again taken for 30 min. Theta burst stimulation (TBS) of the perforant path was then used to induce LTP. The TBS protocol consisted of three sets of 10 trains each, each train consisting of 10 pulses at 100 Hz, at baseline stimulation intensity (inter-train interval: 200 ms; inter-set interval: 1 min). Following TBS, recording at 0.1 Hz continued for 90 min.

Assessment of injection site

After completion of electrophysiological recordings, rats were anesthetized with a pentobarbital overdose and transcardially perfused with 200 ml of 0.9% sodium chloride, followed by 250 ml of 4% paraformaldehyde (4ºC) in 0.01 M phosphate buffered saline (PBS). Brains were removed, post-fixed overnight at 4ºC in the same fixative, and immersed in a 30% sucrose/PBS solutions. Free floating 30 µm coronal sections were collected with a cryostat (Leica, Wetzlar, Germany) in PBS azide 0.05% and stored at 4ºC until use. Sections were mounted on glass slides, let dry and coverslipped with immu-mount media (Thermoscientific, Waltham, and United State). EGFP expression was checked via epifluorescence microscopy (excitation at 480 nm) to validate appropriate transduction in the dentate gyrus for both control- and EphA7

knockdown virus injected rats. Only rats with bilateral infection restricted to the dentate gyrus were included in the electrophysiological and behavioral analyses.

Supplemental references

Andersen, P., Holmqvist, B., and Voorhoeve, P.E. (1966). Entorhinal activation of dentate granule cells. Acta Physiol Scand 66, 448-460.

Jiao, Y., Sun, Z., Lee, T., Fusco, F.R., Kimble, T.D., Meade, C.A., Cuthbertson, S., and Reiner, A. (1999). A simple and sensitive antigen retrieval method for free-floating and slide-mounted tissue sections. J Neurosci Methods 93, 149-162.

Pruss, T., Kranz, E.U., Niere, M., and Volkmer, H. (2006). A regulated switch of chick neurofascin isoforms modulates ligand recognition and neurite extension. Mol Cell Neurosci 31, 354-365.

Richter-Levin, G., and Segal, M. (1991). The effects of serotonin depletion and raphe grafts on hippocampal electrophysiology and behavior. J Neurosci 11, 1585-1596.

Rosenblum, K., Maroun, M., and Richter-Levin, G. (1999). Frequency-dependent inhibition in the dentate gyrus is attenuated by the NMDA receptor blocker MK-801 at doses that do not yet affect long-term potentiation. Hippocampus 9, 491-494.

Sloviter, R.S. (1991). Feedforward and feedback inhibition of hippocampal principal cell activity evoked by perforant path stimulation: GABA-mediated mechanisms that regulate excitability in vivo. Hippocampus 1, 31-40.

Yarom, O., Maroun, M., and Richter-Levin, G. (2008). Exposure to forced swim stress alters local circuit activity and plasticity in the dentate gyrus of the hippocampus. Neural Plast 2008, 194097.
